# Pego do Diabo (Loures, Portugal): Dating the Emergence of Anatomical Modernity in Westernmost Eurasia

**DOI:** 10.1371/journal.pone.0008880

**Published:** 2010-01-27

**Authors:** João Zilhão, Simon J. M. Davis, Cidália Duarte, António M. M. Soares, Peter Steier, Eva Wild

**Affiliations:** 1 Department of Archaeology and Anthropology, University of Bristol, Bristol, United Kingdom; 2 Instituto de Gestão do Património Arquitectónico e Arqueológico, Lisbon, Portugal; 3 Instituto de Gestão do Património Arquitectónico e Arqueológico, Lisbon, Portugal; 4 Instituto Tecnológico e Nuclear, Lisbon, Portugal; 5 VERA Laboratory, Faculty of Physics, University of Vienna, Vienna, Austria; 6 VERA Laboratory, Faculty of Physics, University of Vienna, Vienna, Austria; University of Wisconsin, United States of America

## Abstract

**Background:**

Neandertals and the Middle Paleolithic persisted in the Iberian Peninsula south of the Ebro drainage system for several millennia beyond their assimilation/replacement elsewhere in Europe. As only modern humans are associated with the later stages of the Aurignacian, the duration of this persistence pattern can be assessed via the dating of diagnostic occurrences of such stages.

**Methodology/Principal Findings:**

Using AMS radiocarbon and advanced pretreatment techniques, we dated a set of stratigraphically associated faunal samples from an Aurignacian III–IV context excavated at the Portuguese cave site of Pego do Diabo. Our results establish a secure *terminus ante quem* of ca.34,500 calendar years ago for the assimilation/replacement process in westernmost Eurasia. Combined with the chronology of the regional Late Mousterian and with less precise dating evidence for the Aurignacian II, they place the denouement of that process in the 37th millennium before present.

**Conclusions/Significance:**

These findings have implications for the understanding of the emergence of anatomical modernity in the Old World as a whole, support explanations of the archaic features of the Lagar Velho child's anatomy that invoke evolutionarily significant Neandertal/modern admixture at the time of contact, and counter suggestions that Neandertals could have survived in southwest Iberia until as late as the Last Glacial Maximum.

## Introduction

In the ongoing debate concerning the emergence of anatomical modernity [1]–[16], the Iberian Peninsula occupies a particular place. Current evidence suggests that, south of the Ebro drainage system, Neandertal populations persisted for several millennia after their disappearance everywhere else. In one model—the “Ebro Frontier” [17]–[22]—this time lag was caused by historically contingent demographic and paleoenvironmental factors, with replacement/assimilation ensuing once such factors ceased to operate and along the same lines as in the rest of Europe. Others have suggested that the Iberian pattern is a byproduct of erroneous dating and insufficient data, creating the illusion of punctuation in a process that would have been characterized by a straightforward East-West gradient [23]–[25].

A basic premise of the “Ebro Frontier” is that the Protoaurignacian and succeeding phases of the Aurignacian technocomplex are a proxy for anatomical modernity. This premise is supported by the Oase fossils [26]–[28], which, even if devoid of an immediate archeological context, document the presence of modern humans in the lower Danube ca.40 ka cal BP (calendar years ago), i.e., in the time interval—ca.40–42 ka cal BP [13], [29]–[32]—of the Protoaurignacian (unless otherwise stated, the calendar chronology used in this study derives from radiocarbon dates calibrated with CalPal [33]–[34]). Moreover, from Bulgaria in the East to the Asturias in the West, no Neandertal fossils have ever been found in the Protoaurignacian, which replaced a diverse array of Neandertal-associated, so-called Transitional technocomplexes (e.g., Szeletian, Uluzzian, Châtelperronian) [13], [31], [35].

Bearing in mind that (1) the Protoaurignacian and the Aurignacian I remain unknown in southwest Iberia, (2) secure Mousterian occurrences well dated to the 37–42 ka cal BP time interval exist in Murcia, Gibraltar and Portugal, and (3) at the Sima de las Palomas (Murcia), such contexts contain a large number of fragmentary but diagnostically Neandertal skeletal remains, the southwest Iberian Neandertal persistence pattern is hard to deny [36]–[40]. Therefore, current debates focus on the duration of the pattern. The “Ebro Frontier” position has been that it lasted for about five millennia and disappeared no later than ca.35 ka cal BP, as implied by (1) the southwest Iberian sites with diagnostic Aurignacian II and III–IV assemblages and (2) the fossil localities spreading from Romania to France that directly associate these younger stages of the Aurignacian with modern humans [4]–[6], [41]–[44]. Others propose that the earliest Upper Paleolithic of the area is the Middle Gravettian, implying a regional survival of the Middle Paleolithic (and Neandertals) until the ca.28–32 ka cal BP interval [45]–[48], if not the Last Glacial Maximum [49] [contra: 22,50]. This chronostratigraphic debate is also ancillary to the interpretation of the Neandertal-like features in the anatomy of the 30,000 year old Lagar Velho modern human child—as indicating admixture at the time of contact, or as the byproduct of an isolated hybridization event [51]–[53].

**Figure 1:**
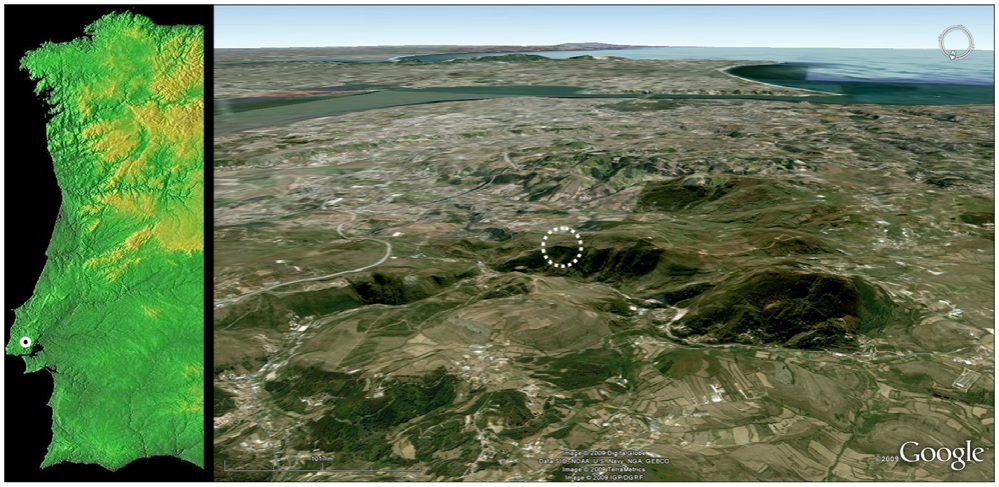
Pego do Diabo: location and geographical setting. Latitude: 38°51′47″N. Longitude: 009°13′13″W. Elevation: 250 m. The dotted circle in the GoogleEarth view (where elevations are 1.5× and the site is seen from NNW, with the city of Lisbon and the estuary of the Tagus to the south of it) indicates the limestone ridge where the cave opens.

**Figure 2:**
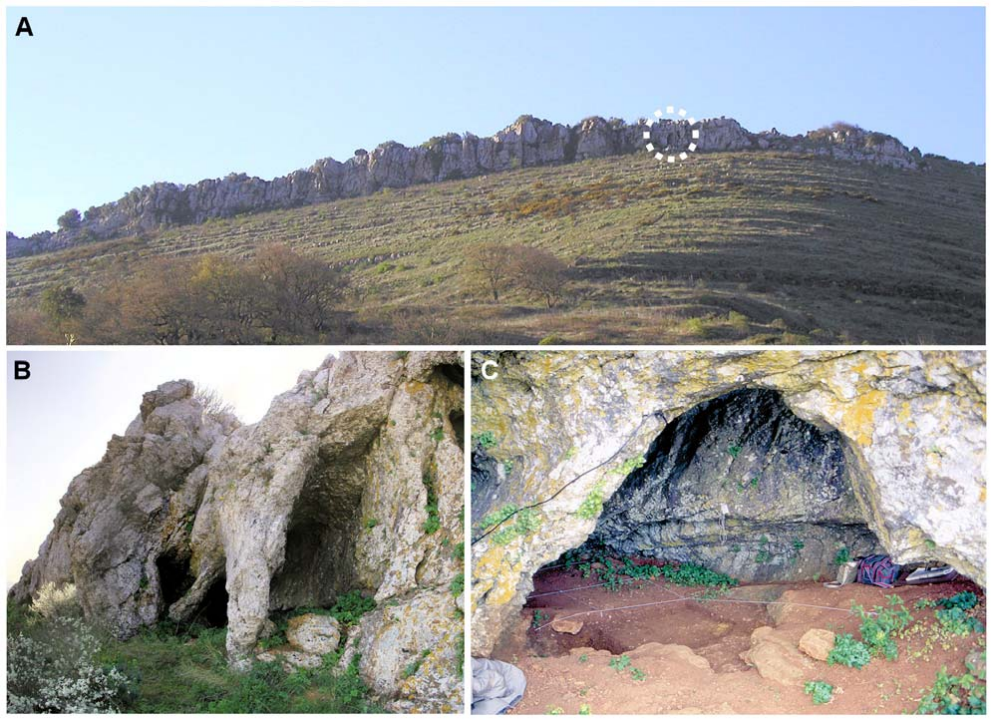
Pego do Diabo: the site. **A**. View over the Pego do Diabo limestone ridge (the dotted circle marks the general area where the entrance is located). **B**. The cave entrance. **C**. The porch area in 1988. Rows 5–7 of the grid are visible in the foreground. In this area, the fill was very shallow, and its surficial part probably included scattered material derived from 1960s backdirt and sieving residues.

The site of Pego do Diabo (Pool of the Devil), a small, North facing limestone cave in the outskirts of Lisbon (Figures 1 and 2), plays a key role in all of this, as it yielded a small assemblage of Dufour bladelets—an index fossil of the Aurignacian technocomplex—associated with a conventional radiocarbon date of ca.32.8 ka cal BP [41], [54]. This evidence, however, has been questioned on several grounds: the putative non-diagnostic nature of the Dufour bladelet tool type; the proposition that other tools from the site are of Gravettian affinities; the presence of intrusive elements in the fauna; and the potential inhomogeneity of the bulk bone samples used for dating [55]. In order to address these concerns, we restudied the faunal assemblage, undertook an extensive program of AMS radiocarbon dating of single item samples, and conducted a comparative revision of the bladelet tools.

## Results

### Previous Work

**Figure 3:**
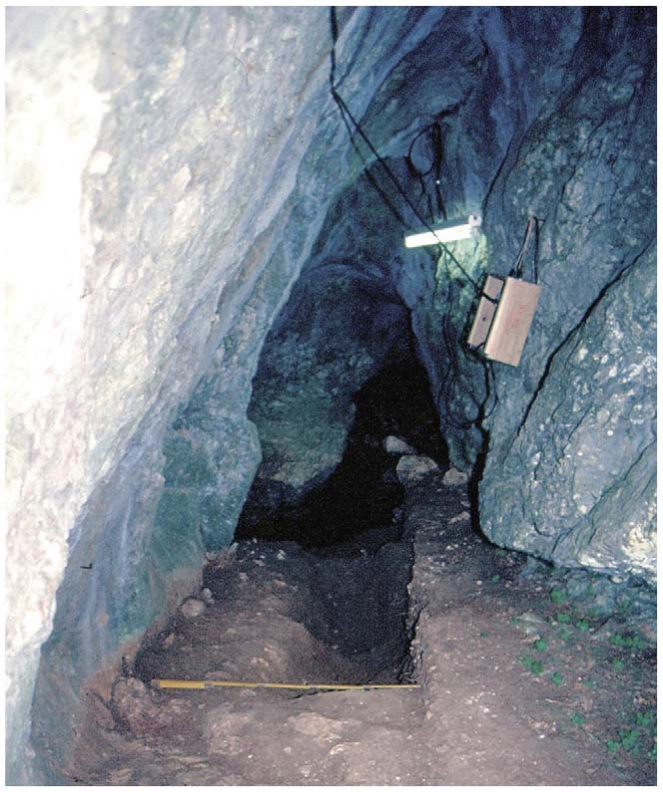
Pego do Diabo: interior view during the 1989 excavation work. The 1960s trench is just beyond the lit area. In the foreground, note the large bioturbation feature (possibly a badger burrow) in rows 8–10 of the grid.

**Figure 4:**
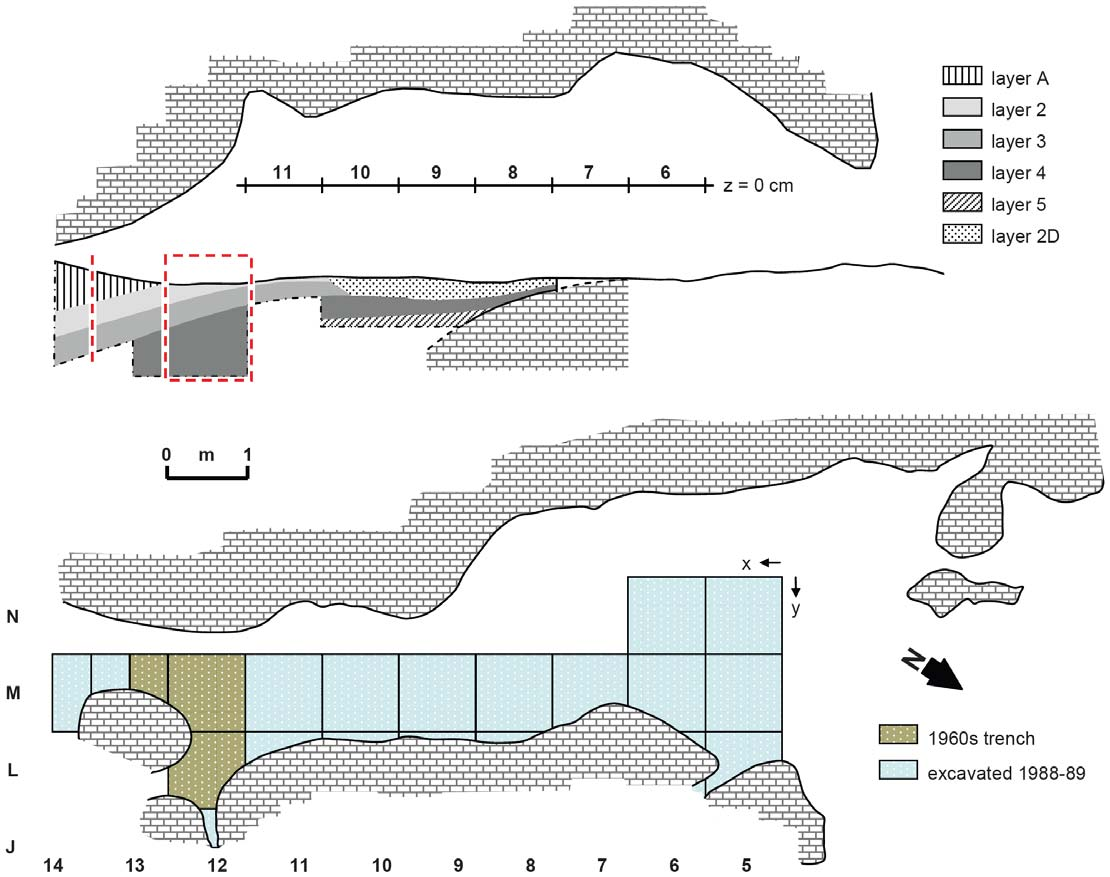
Pego do Diabo: plan and profile of the cave. The position of the profile photos in Figure 5 is indicated by the dashed red lines. Given its overall shape, the geometry of the bedrock, and the direct exposure to the strong N-NW winds that blow in the valley, it was only in the reduced area where the 1960s trench was placed that humans could have found some shelter in the cave, explaining the ephemeral nature of the Paleolithic occupations.

**Figure 5:**
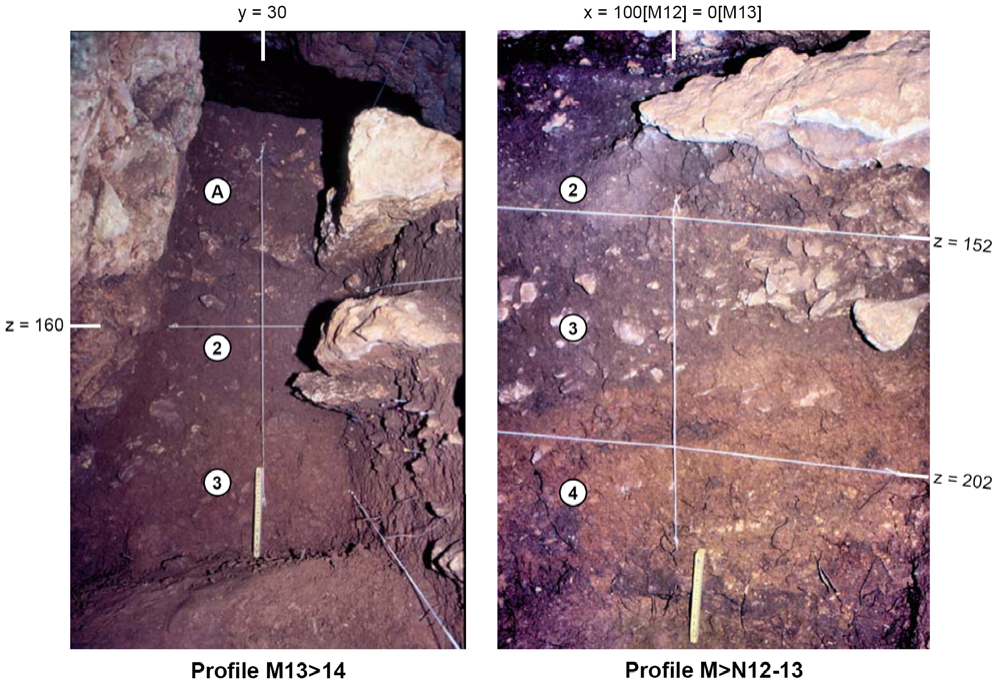
Pego do Diabo: the stratigraphy at the back of the cave. The photos were taken at the end of the 1988 field season. Fac-simile reproductions of the field drawings are provided in Figures S1, S2. Layer 2 is a “cave earth” deposit sandwiched between two episodes of roof collapse that generated the thick slabs visible in the profiles. Layer 3 has a major silt/clay component and features small limestone clasts with surface weathering and manganese staining, suggestive of pedogenesis and agreeing with the discontinuous interface to suggest a depositional hiatus prior to the accumulation of layer 2. Layer 4 is a compact, nearly sterile red clay deposit traversed by roots and where small bioturbation features are readily apparent. In the M13>14 profile, the narrow band of clay-enriched sediments visible along the cave wall from top to bottom of layer 2 denotes percolation from the surface.

Pego do Diabo (a.k.a. Loca do Gato—Cat's Den), was first explored between 1965–66, when the Department of Palethnology of the Portuguese Speleological Society visited the cave and observed no trench (Carl Harpsøe, personal communication), and 1973, when the existence of a trench was first reported [56]. The profiles left by the unknown excavators of this trench guided subsequent excavation work, carried out over two seasons in 1988–89 [41], [54] (Figures 2, 3, 4, 5). Recognized to a depth of ca.1.4 m, the fill comprised, from top to bottom, six different units (Figures 4, 5, S1, S2). Layer A, only present at the back of the cave, is a localized cone accumulated under a roof chimney in later Holocene times. The squares in row 11 of the grid, immediately west of the 1960s trench, featured a thin surficial level formed of disturbed Pleistocene sediments that also contained some recent material (layer 1), under which there was a broadly intact Pleistocene sequence comprised of four different units (layers 2–5).

Towards the entrance, in rows 5–10 of the grid, the Pleistocene sequence was very shallow and its surface formed the floor of the cave. As a result of trampling by animals and humans, coupled with bioturbation (in some areas, extending all the way down to bedrock; Figure 3), intrusive material (small, often round-edged sherds of wheeled and glazed pottery, as well as sheep/goat teeth) was introduced in deposits of layer 2 that primarily contained Pleistocene bone and lithics (these disturbed deposits are referred to as layer 2D) (Table 1). In grid units J-M/11-12, such recent Holocene intrusions were scarce and limited to the uppermost spit of layer 2, while significant bioturbation features were restricted to areas along the north wall of the cave, where disturbance was compounded by the “wall effect” phenomenon, a common affliction of most cave and rock shelter sites (Figure S3).

**Table 1:** Pego do Diabo: vertical and horizontal distribution of ceramic sherds (1998–89 excavations).

Squares M13-14 (a)		Squares J-M/11-12(b)		Squares M-N/5-10 (b)	
Stratigraphic Unit	N	Stratigraphic Unit	N	Stratigraphic Unit	N
Layer A	16	Layer 1	25	Layer 2D	211
Layer 2 upper	2	Layer 2 upper	1	Layer 3	–
Layer 2 lower	–	Layer 2 lower	–	Layer 4	–
Layer 3	–	Layer 3	–	–	–
Layer 4	–	Layer 4	–	–	–

(a) Five possibly Iron Age, the other Medieval and/or post-Medieval.

(b) Medieval and/or post-Medieval.

As all diagnostic intrusions were of a recent Holocene chronology, the original study of the site assumed that all the lithics recovered in layers 1 and 2D related to the human occupation documented by the assemblage recovered *in situ* in layer 2 of rows 11–12 (no lithics were found in squares M13-14). The corresponding inventory is given in Table 2. The retouched tool assemblage is dominated by microliths that unambiguously fit the consensus definition of the Dufour bladelet [57]–[58], although one was classified separately because the retouch is bilateral-direct; of these seven pieces, four came from undisturbed areas in grid units L/M11 and in the M12>11 profile.

**Table 2:** Pego do Diabo: lithics from layers 2 and 2D (a).

Artifacts	Flint	Quartz	Total
DEBITAGE AND DEBRIS			
Flakes	6	6	12
Chips	6	3	9
FORMAL RETOUCHED TOOLS			
Atypical endscraper on flake (b)	1	–	1
Atypical borer	1	–	1
Sidescraper (b)	1	–	1
Dufour bladelet	6	–	6
Bladelet with marginal, direct, bilateral retouch	1	–	1
Retouched tool fragment	1	–	1
TOTAL LITHICS	23	9	32

(a) After [41], [54].

(b) In layer 1 (i.e., the disturbed, uppermost part of the Pleistocene sequence in squares L-M/11 and J12), and assumed to derive from layer 2.

The assumption concerning the stratigraphic provenience of the lithics from layers 1–2D was further warranted by the fact that layer 5 was sterile and layers 3 and 4 yielded very few artifacts: one flint flake in layer 4, and six in layer 3, which also contained one quartz and one flint chip. The patina and surface condition of these flints (Figure S4) suggest that they derive (through natural formation processes) from the kinds of Lower and Middle Paleolithic surface scatters that are ubiquitous in the clayey slope deposits of the Tertiary “basaltic cover” capping the regional geology above and beyond the Pego do Diabo limestone ridge [59]. Such distinctively patinated material was recovered neither in layers 1–2 of rows 11–14 nor in the disturbed deposits of rows 5–10.

A similar assumption was made in the original faunal study [60]–[61], although a third component was in this case added. Besides the bones found *in situ* in squares L-M11 and the bones from layer 1 and from the disturbed deposits in rows 5–10 judged to be of Pleistocene age (on the basis of taxonomy and mineralization, i.e., to the exclusion of sheep/goat and the fresh, clearly intrusive bones of rabbits and other small animals), the “layer 2” category of that study also included the faunal material of Pleistocene age (inferred on the basis of identical criteria) accumulated at the bottom of the 1960s trench as a result of the gradual collapse of its profiles. The addition of this ensemble (labeled REM, from the Portuguese *remeximento*, or disturbance) was justified, as (a) layers 3 and 4 were rather poor in faunal remains, (b) most *in situ* bone finds from layers 3 and 4 presented extensive manganese staining (Figure S5), a feature shared neither by those from layer 2 nor by those in the REM ensemble (c) the significant amount of clay in the sedimentary matrix of layers 3 and 4 implied that, at the corresponding elevation, the profiles degraded much less than at the elevation of the more typical layer 2 “cave earth” deposit, and (d) the addition of the REM ensemble increased by >50% (from 864 to 1336) the number of identified specimens (NISP) for layers 1–2, thus multiplying its potential for interpretation and comparison.

Although this amalgamation was unlikely to affect the overall structural properties of the stratigraphically *in situ* layer 2 bone assemblage (and in fact did not; see below), the possibility could not be excluded that, in the process, residual components of a different age had become incorporated in the expanded sample. The selection of material for conventional radiocarbon dating in the laboratory of the Instituto Tecnológico e Nuclear (Sacavém, Lisbon), carried out in 1989, bore this possibility in mind. Accordingly, for layer 2, only squares L-M11 were used, and, as Holocene intrusions had been identified in their upper spits during excavation, separate samples were submitted for the upper (2a) and lower (2b) parts of the layer, in order to identify/isolate the potential effect of contaminants in the sample from 2a. Layer 3, in turn, was sampled from squares M13-14, an area where layer 2 was almost sterile and, therefore, the possibility of contamination of the layer 3 faunal assemblage by later material (e.g., as a result of decapage error) could be excluded.

The results obtained are presented in Table 3. The date for layer 2 of the M13>14 profile must relate to the Iron Age pottery recovered in overlying layer A, reflecting percolation from above, along the cave wall, of the dated charcoal flecks (Figure 5). The date for layer 3 was obtained from rather impure collagen, suggesting incomplete decontamination, and the dating lab advised that the result be treated as a minimum age only. Given the homogeneous artifact content of layer 2, the discrepancy between the samples for 2a and 2b was in turn considered to be a byproduct of the residual presence of recent Holocene material in the 2a bulk bone sample. The ICEN-732 result for 2b was therefore retained as dating the Aurignacian occupation of the site to ca.28.1 ka ^14^C BP (31.3–34.2 cal BP, at the 95% confidence level) [54].

**Table 3:** Pego do Diabo: conventional radiocarbon results (a).

Sample ID	Layer	Composition	Lab #	Age [^14^C years BP]	cal BP age	δ^13^C [‰]
M13 (b)	2	Unidentified wood charcoal	ICEN-306	2400±80	2800-2240	−25.0
M11sc37+L11sc2 (c)	2 (spit 2a)	Large mammal shaft fragments (300 g)	ICEN-490	23,080±490	–	−19.1
M11sc38+L11sc31	2 (spit 2b)	Large mammal shaft fragments (395 g)	ICEN-732	28,120+860/−780	34,200-31,320	−20.7
M13sc16/17+M14(1989) (d)	3	Large mammal bone fragments and rabbit bones (635 g)	ICEN-491	18,630±640	–	–

(a) Beta counting, standard gelatin production. All uncertainties in radiocarbon age results are 1σ. Calibration used CalPal with the CalPal_2007_HULU calibration curve [33]–[34], and cal BP results are the 95.4% probability age ranges.

(b) Sampled from south profile of M12, where charcoal from overlying Holocene level A percolated downwards along the wall (Figure 5).

(c) Spit 2a contained scant Holocene intrusions.

(d) Unreliable result; the collagen was impure (32% of residue after combustion), the yield low, and δ^13^C could not be measured due to an accident in the mass spectrometer; counts for the rabbit bone component are given in Table S1.

Alternatively, it has been suggested that the ca.23.1 ka ^14^C BP result dated layer 2 to the Gravettian and that the ca.28.1 ka result reflected sample contamination by older Mousterian bones derived from underlying layer 3 [55]. This alternative view of the evidence is inconsistent with the exponential nature of radiocarbon decay [41]: for a sample with an expected age of ca.23 ka ^14^C BP to yield an age of ca.28 ka ^14^C BP, the older “contaminating” fraction would have to outweigh the “genuine” one in a proportion of 4∶1. Moreover, at Pego do Diabo, (a) layer 3 contained four and a half times fewer bones than layers 1–2, and (b) no independent Mousterian contamination was apparent in the lithics from layers 1–2, as no flints with the distinctive layer 3 patina were found therein.

A more pertinent objection to the dating of layer 2 to ca.28.1 ka ^14^C BP is that conventional bone results for the period of the Middle-to-Upper Paleolithic transition in Europe tend to be in disagreement with overall chronostratigraphic patterns and, when compared with results obtained on charcoal or with the AMS technique, appear systematically rejuvenated, often by many millennia [25], [29]–[30]. The differences are likely due to insufficient removal of contaminants—a particular problem with conventional methods (because of the larger sample sizes required), but one that can also affect AMS dating (as demonstrated by differences in ages produced by different laboratories using different treatments on a single sample) [62]. Rejuvenation thus had to be considered as a problem at Pego do Diabo too—the Aurignacian occupation at this site could conceivably be much older, with implications for the pattern of delayed Neandertal survival in southwest Iberia. The site therefore needed to be redated with methods that benefited from recent technical advances in sample pretreatment and measurement precision [63]–[64].

### The Faunal Assemblage

In the framework of the new dating project, we revisited the site's faunal assemblage with four objectives in mind: (a) to identify anthropically modified bones whose dating could establish the times of human activity in the cave independently of post-depositional disturbance; (b) to study species composition and age-at-death profiles from subsets that excluded the material from all disturbed, or potentially disturbed proveniences; (c) to select from these subsets suitable material for dating by association; (d) to compare such “pure” subsets with assemblages from well stratified sites in order to derive chronological patterns that could assist us in the interpretation of the radiocarbon dating.

Our results are presented in Tables 4, 5, 6. The samples from layers 3 and 4 are too small to allow sensible quantitative conclusions. In layer 3, the abundance of rabbit remains is striking. If we also consider the bone consumed in the dating of radiocarbon sample ICEN-491 (Table S1), rabbits correspond, in NISP terms, to 83% of the fauna (compared to only 32% in the amalgamated “layer 2” sample used in the 2000 faunal study). The Pego do Diabo rabbit bones exhibit punctures typical of small carnivores, while no cutmarks or long bone cylinders, typical of human consumption, were observed [60]. Lynx and fox are the only carnivores present in the assemblage, lynx feeds primarily on rabbits, and therefore this evidence suggests that, in layer 3 times, the back of the cave was used as a lynx den.

**Table 4:** Pego do Diabo: bone and tooth counts per stratigraphic unit (a).

		This study				2000 study	
Taxa		4	3	2	1/2D	3	1/2D/2/REM
AMPHIBIA							
Toad	*Bufo*	–	–	1	1	–	–
Other amphibians		–	–	–	5	–	–
REPTILES							
Tortoise	*Testudo*	–	1	–	–	–	–
Lizard		–	–	–	1	–	–
BIRDS							
Galliforme	cf. *Gallus*	–	–	–	1	–	–
Partridge	*Alectoris*	–	–	1	1	–	–
Goose	*Anser*	–	1	–	–	–	–
Crow	*Corvid*	–	–	1	–	–	–
Passerines		–	–	–	1	–	–
Other birds		–	–	2	1	–	–
MAMMALS							
Bat		–	–	1	5	–	–
Shrew	*Talpa*	–	–	–	2	–	–
Dormouse	*Eliomys*	–	–	–	1	–	–
Field mouse	*Apodemus*	–	–	1	–	–	–
Vole	*Microtus*	–	–	1	–	–	–
Aurochs/cattle	*Bos*	–	–	–	2	–	–
Sheep/Goat/Ibex	*Ovis/Capra*	–	–	1	4	–	–
Ibex	*Capra*	–	–	1	2	–	29
Ibex/Chamois	*Capra/Rupicapra*	–	–	–	–	1	42
Chamois	*Rupicapra*	–	–	1?	1?	–	22
Sheep (b)	*Ovis*	–	–	–	1	–	–
Red deer	*Cervus*	–	1	25	11	1	197
Roe deer	*Capreolus*	–	–	–	1?	–	–
Wild boar/pig	*Sus*	–	–	–	2	–	12
Equids	*Equus*	–	–	4	3	3	73
Hare	*Lepus*	–	–	1	–	1	10
Rabbit I	*Oryctolagus*	21	41	32	70	337	707
Hyaena (d)	*Crocuta*	–	(2)	1	(2)	–	6
Bear	*Ursus*	–	–	1	1	–	3
Badger	*Meles*	–	–	–	6	–	6
Leopard	*Panthera*	–	–	1?	–	–	–
Lynx	*Lynx*	–	–	3	5	2	28
Wildcat	*Felis silvestris*	–	2	–	–	–	–
Wolf	*Canis*	–	–	1?	1	–	16
Fox	*Vulpes*	–	1	2	–	1	15
Unidentifiable chips (e)		20	163	81	526	141	1022
Semidigested large mammal bones		–	1	1	11	–	–
Gnawed mammal bones		–	–	1	2	–	–
Cutmarked mammal bones		–	2	–	1	–	–

(a) Counts in this study are the ‘Parts of the Skeleton Always Counted’ (PoSACs; [65], [99]). Counts in the 2000 study are NISP (Number of Identified Specimens) for the total assemblage. In the 2000 study, the material from layers 1, 2, and 2D, as well as from REM (material from profile collapse recovered from the bottom of the 1960s trench) was grouped in a single “Layer 2” assemblage [61]. In this study, layer 2 includes only *in situ* material, while the material from disturbed areas was combined with layer 1 to form the “1/2D” sample.

(b) As trace intrusive material from the recent Holocene was found in the upper part of layer 2 (Table 1), this one sheep must represent a specimen that went undetected when the collection was sorted to exclude such contaminants.

(c) Rabbit counts for layer 3 do not consider the material included in the bulk radiocarbon sample ICEN-491 (Table S1).

(d) Hyena counts between parentheses refer to coprolites.

(e) “Chips” are unidentifiable fragments of large mammal bones. These counts do not consider the material included in the bulk bone samples ICEN-490, ICEN-491 and ICEN-732 (Table 3).

**Table 5:** Pego do Diabo: age at death of red deer, equid and aurochs in layer 2 (a).

Bone/Tooth	Fusion state	Red Deer	Equid	Aurochs
BONE				
Scapula (glenoid)	Unfused	–	–	–
	Fused	1	–	–
Distal humerus	Unfused	1 metaphysis	–	–
	Fused	–	–	–
Distal radius	Unfused	1 metaphysis	–	–
	Fused	–	–	–
Distal metacarpal	Unfused	–	1 metaphysis	–
	Fused	–	–	–
Distal tibia	Unfused	1 metaphysis ( = newborn)	–	–
	Fused	–	–	–
Astragalus	n.a.	3 (2 = newborn)	–	–
Calcaneum	Unfused	1	–	–
	Fused	–	–	–
Proximal phalanx	Unfused	2 epiphyses, 3 metaphyses (all newborn)	–	–
	Fused	1	–	–
Middle phalanx	Unfused	1 epiphysis, 1 metaphysis	–	–
	Fused	5	–	–
Distal phalanx	n.a.	3 (1 = newborn)	1 (juvenile)	–
Metapodial halves	Unfused	2 epiphysis (1 = newborn)	1 metaphysis, 1 epiphysis	–
	Fused	1	–	–
TOOTH				
Lower dP3		5 (3 = newborn)	–	1 (fairly well worn)
Lower dP3/dP4		–	3 (2 = few weeks old)	–
Lower dP4		1	–	–
Lower M1/2		2 (1 = newborn)	–	–

(a) Based on mandibular teeth and limb-bones with age information (i.e. deciduous or adult teeth, or state of fusion of the epiphyses). Cases where a tooth or bone is estimated to be derived from a newborn animal (i.e., enamel shows little wear, or the bone is poorly ossified) are listed in parentheses. Other estimates of age or degrees of tooth wear are also given where possible. Bones and teeth normally recorded (such as pelves, femora and mandibular P4s, M1s, M2s and M3s) do not appear in this table because nore were found at Pego do Diabo.

**Table 6:** Pego do Diabo (PGD) vs. Gruta do Caldeirão (CAL): taphonomic indicators.

	PGD-2	PGD-1/2D	CAL	CAL	CAL	CAL
Index/Measure	Aurignacian	Aurignacian	Mousterian	EUP	Solutrean	Magdalenian
Mammal bones (excluding rabbits) (a)	42	39	122	116	267	175.5
Large carnivores [%] (b)	11	7	10	14	3	3
Juveniles among red deer [%]	66	–	68	69	32	16
Juveniles among equids [%]	100	–	70	77	14	0
Ungulate to rabbit [ratio] (c)	1	0.4	0.6	0.4	0.1	0.04
“Chips” to “identified” [ratio] (d)	4	21	8	9	11	23
Semidigested bones [%] (e)	2	1	16	7	2	<1

(a) These counts are “PoSAC” [65], [99].

(b) The percentage of large carnivores is calculated by dividing the number of large carnivore (i.e., hyaena, bear, lion, leopard and wolf) bones by the total number of mammal herbivore and large carnivore bones.

(c) Caldeirão rabbit numbers after [66].

(d) The ratio “chips” to “identified” is calculated by dividing the number of unidentified chips plus the number of ungulate bones and teeth by the number of ungulate bones and teeth. Note that the values for Pego do Diabo, and especially for the “PGD-2” sample, are affected by the sacrifice of a significant proportion of the “chips,” prior to analysis, for conventional radiocarbon dating.

(e) The percentage of semidigested bone considers the total of identified large mammals, rabbits and fragments. As with the values for the “chips” to “identified” ratio, this index may be affected, at Pego do Diabo, by the sacrifice of significant numbers of unidentified bone fragments, prior to analysis, for conventional radiocarbon dating.

Where layer 2 is concerned, the *in situ* assemblage features a composition identical to that from disturbed proveniences (1/2D), and the combined range of species, as well as their relative abundances, is broadly the same as in the 2000 study, despite the taxonomic reassignment of certain bones. The diversity is high (Figure S6), and, apart from the omnipresent rabbit, the mammal fauna is dominated by red deer. Equids and ibex are also common, while other large mammals like aurochs, wild boar and chamois occur in smaller numbers. The ibex is presumed to be *Capra pyrenaica*, and two equid species are probably present: the small one includes a pointed terminal phalanx similar to *E. hydruntinus*, while an upper cheek tooth has an elongated protocone similar to that in *E. caballus*. The most striking aspect of the large herbivore remains is the scarcity of teeth and bones from adults (Table 5). Of the 35 red deer teeth and bones counted, only 12 are derived from older animals, making the percentage juvenile red deer represented approximately 66% (Figures S7, S8). The layer 2 fauna also includes a wide spectrum of carnivores, which represent a significant proportion of the mammal bones (11% of the *in situ* assemblage).

There were no cutmarked bones among the faunal remains from *in situ* layer 2, and only one in layer 2D (which turned out to be of medieval age when directly dated; see below). The number of gnawed bones is also low (Table 4). These observations concur with previous diagnoses of the fauna as primarily non-anthropogenic [54], [60]–[61], and indicate that hyenas, although present in the assemblage, may have played a marginal role in its accumulation and modification. Several partially digested rabbit bones suggest that, as in layer 3, lynx must have been responsible. However, the presence of semidigested bones of larger mammals (including a proximal phalanx of bear, a red deer astragalus, two red deer second phalanges and a red deer metapodial condyle) implicates a large bone eating carnivore, probably the wolf. Large felids may also have contributed to the accumulation, and leopard has been tentatively identified.

When compared with the reference assemblages from Gruta do Caldeirão [65]–[66] (Tables 6, S2), the fauna from layer 2 of Pego do Diabo falls very definitely among the earlier periods represented in that sequence—Mousterian and Early Upper Paleolithic—in such parameters as percentage of large carnivores, percentage of juveniles among red deer and equids, and ungulate to rabbit ratio. The “chips” to “identified” ratio is more difficult to interpret, as the assemblages from Pego do Diabo are small, and the sacrifice of a significant number of “chips” for radiocarbon dating, prior to analysis, means that the values in Table 6 are underestimates. However, doubling the value for the *in situ* sample would still leave it in the range of the Caldeirão basal levels, while the higher value for the sample from disturbed layer 2D may reflect the effects of continued post-depositional attrition and trampling.

The features that layer 2 of Pego do Diabo shares with the basal levels of the Caldeirão sequence—high level of taxonomic diversity, carnivores as the primary accumulators, abundance of large carnivore taxa—are replicated in all other known late Mousterian and Early Upper Paleolithic faunal assemblages from Portugal [61], [65], [67]–[68]. These assemblages reflect a time when competition for the use of small, narrow caves of this kind had not yet tipped in favor of humans. In the Gruta do Caldeirão sequence, this watershed is crossed in layer I, dated to 22,900±380 ^14^C BP (OxA-1940) [54], [65]. From its broader context we can thus conclude that the fauna from layer 2 of Pego do Diabo constrains the age of the associated artifact assemblage to >27,500 calendar years ago.

### The AMS Radiocarbon Dating

**Figure 6:**
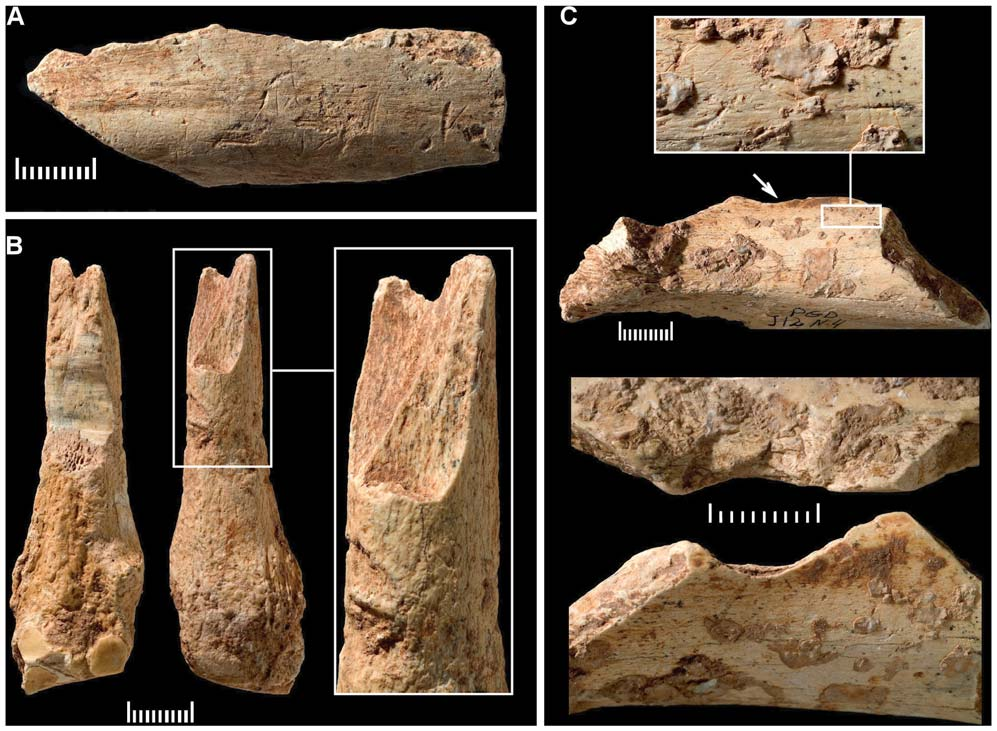
Pego do Diabo: the AMS-dated, anthropically modified bone samples from layers 3 and 2D. **A**. M10, layer 2D, cutmarked; **B**. L11-6, layer 2/3, broken fresh, and with percussion marks (the scraped area was sampled for assessment of the carbon and nitrogen contents prior to submission); **C**. J12-4, layer 2/3, cutmarked and with a percussion break (indicated by the arrow). All scales are in mm. For dating results, see Table 7.

**Figure 7:**
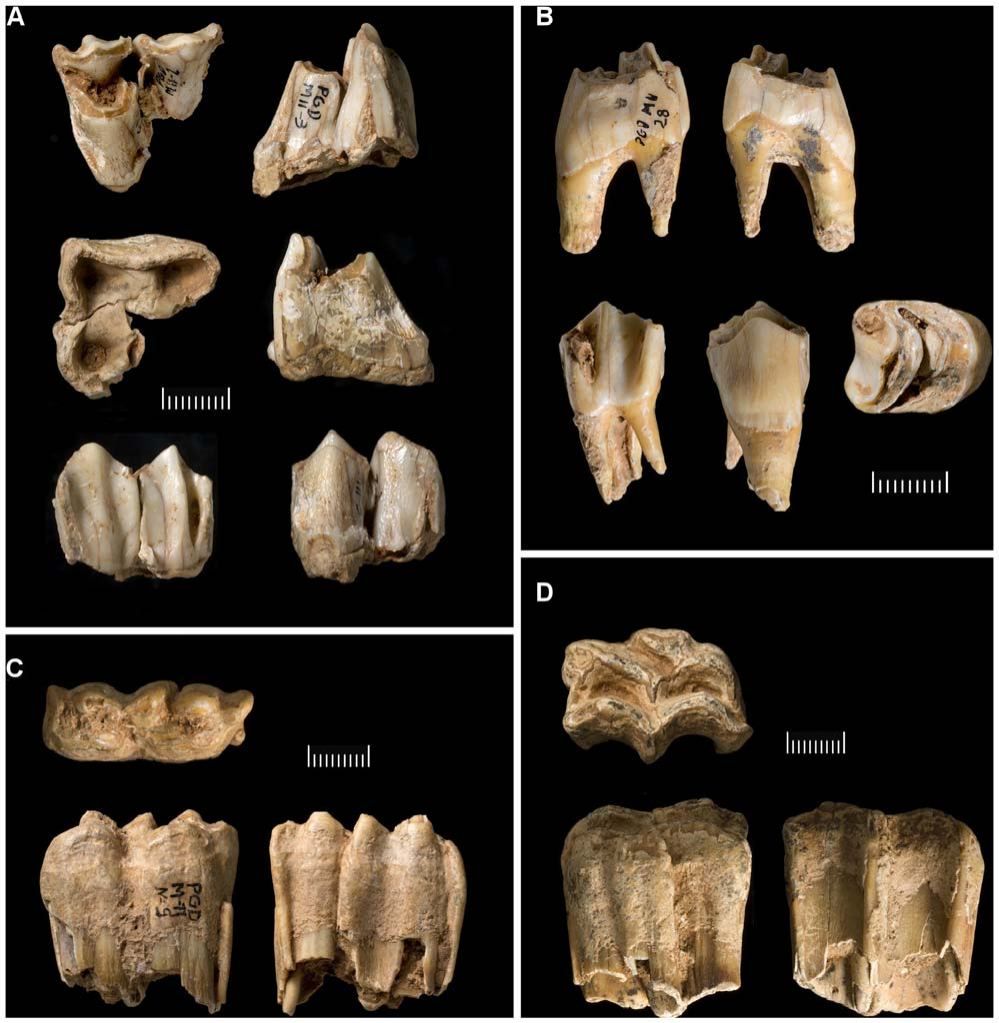
Pego do Diabo: the AMS-dated tooth samples from layer 2 stratigraphically associated with the Aurignacian lithics. **A**. M11-2/3, red deer upper molar; **B**. M11-28, red deer upper premolar; **C**. M11-9, equid deciduous molar; **D**. M11-24, equid deciduous upper molar. All scales are in mm. For dating results, see Table 7.

**Figure 8:**
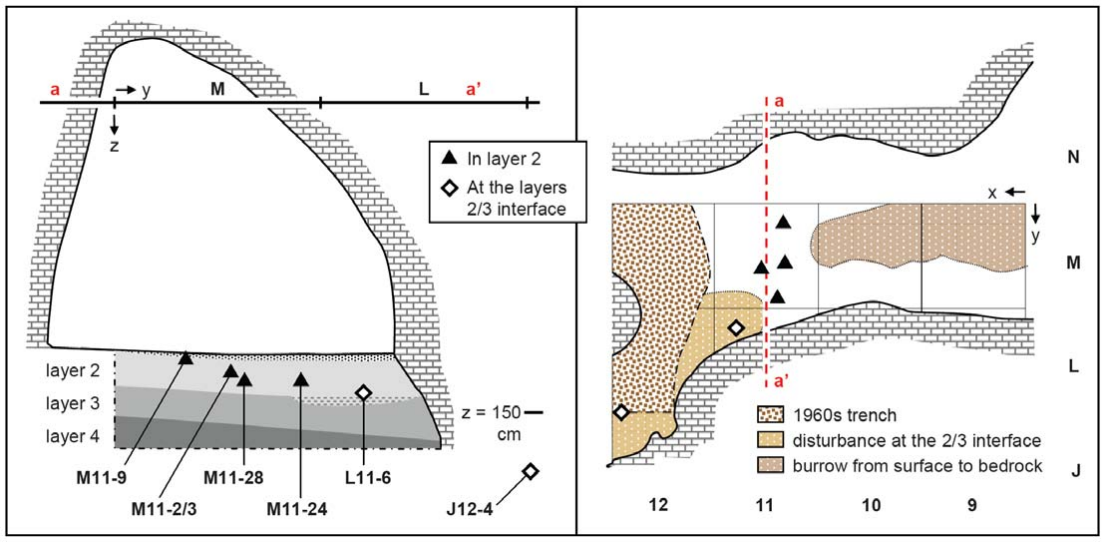
Pego do Diabo: projection on plan and profile of the piece-plotted, AMS-dated samples. Samples L11-6 and J12-4, both from a band of somewhat disturbed deposits at the interface between layers 2 and 3, probably derive from the latter, and were in any case deeper than the others. Their older age is in agreement with their stratigraphic position. The cutmarked bone of medieval age found in layer 2D of square M10 was not piece-plotted at the time of excavation.

For the successful samples (Figures 6, 7), provenience plots are given in Figure 8, and dating results, together with details of sample chemistry, in Table 7. A list of the failed samples (Figures S5, S7, S8) can be found in Table S3.

**Table 7:** Pego do Diabo: AMS radiocarbon results obtained for the samples in Figures 6, 7 (a).

Sample	Layer	Composition	Lab #	Age [^14^C years BP]	cal BP age	Used [g]	Yield [mg]	Yield [%]	C [%]	C∶N [ratio]	δ^13^C [‰]	δ^15^N [‰]
ANTHROPICALLY MODIFIED BONES												
M10 (b)	2D	Shaft fragment	OxA-15005	1353±25	1340-1260	0.90	24.0	2.7	47	3.2	−20.3±0.2	–
L11-6 (c)	2/3	Equid metacarpal	OxA-15499	34,900±1000	42,240-37,320	0.82	5.4	0.7	53	3.3	−20.9±0.2	5.5±0.1
			OxA-X-2272-25 (d)	35,050±750	–	1.99	7.5	0.4	36	3.3	−20.1±0.2	–
J12-4 (c)	2/3	Shaft fragment	OxA-15004	38,750±650	43,820-41,860	0.84	14.7	1.7	46	3.2	−19.8±0.2	–
STRATIGRAPHICALLY ASSOCIATED TOOTH SAMPLES												
M11-2/3	2 (spit 2a)	Upper molar (*Cervus elaphus*)	VERA-4050	30,260+330/−320	34,990-33,950	1.3	10.8	0.8	35 (f)	3.0 (f)	−20.4±1.7 (g)	6.8 (f)
											−20.0 (f)	
			VERA-4050UF1B	30,290+320/−310	–	1.8	3.8	0.2	–	–	−22.1±0.7 (g)	–
			VERA-4050UF2B	28,820+270/−260	–	–	2.6	0.1	–	–	−23.8±0.7 (g)	–
M11-9	2 (spit 2a)	Deciduous molar (*Equus* sp.)	VERA-4049	29,810+310/−300	34,660-33,540	1.3	8.3	0.6	38 (f)	3.1 (f)	−14.2±6.1 (g)	8.1 (f)
											−20.4 (f)	
			VERA-4049UF1B	Failed	–	–	–	–	–	–	–	–
			VERA-4049UF2B	29,640+320/−310	–	1.8	7.8	0.4	–	–	−19.2±0.8 (g)	–
M11-24	2 (spit 2b)	Deciduous upper molar (*Equus* sp.)	VERA-4048 (e)	28,320+280/−270	33,440-32,080	1.3	14.5	1.1	36 (f)	3.1 (f)	−19.2±0.9 (g)	7.6 (f)
											−20.6 (f)	
			VERA-4048B	27,290±230	–	1.5	4.2	0.3	–	–	−18.6±0.9 (g)	–
			VERA-4048C	27,740±230	–	1.8	15.1	0.8	–	–	−21.6±0.7 (g)	–
			VERA-4048UF1B	28,040±250	–	1.8	2.9	0.2	–	–	−22.6±0.7 (g)	–
			VERA-4048UF2B	Failed	–	–	–	–	–	–	–	–
M11-28	2(spit 2b)	Upper P4 (*Cervus elaphus*)	VERA-4047	29,090±270	34,250-32,850	1.3	11.4	0.9	33 (f)	3.0 (f)	−19.2±1.3 (g)	6.7 (f)
											−20.2 (f)	
			VERA-4047UF1B	29,150+280/−270	–	1.3	7.1	0.5	–	–	−19.8±0.6 (g)	–
			VERA-4047UF2B	28,360+270/−260	–	–	6.8	0.5	–	–	−19.9±0.5 (g)	–

(a) Pretreatments: OxA, ultrafiltration; VERA, standard gelatine production and ultrafiltration (UF1 results – gelatin fraction >30 kDa; UF2 results – gelatin fraction <30 kDa). All uncertainties in radiocarbon age, δ^13^C and δ^15^N results are 1σ. Calibration used CalPal with the CalPal_2007_HULU calibration curve [33]–[34] , and cal BP results are the 95.4% probability age ranges.

(b) In area where level 2 was surface and where a large burrow disturbed the deposits down to bedrock (see Figures 3, 4).

(c) In areas at the interface between levels 2 and 3, against the north wall of the cave, described in field documents as clayey, with many roots, and featuring an apparent mix of bone material from both units (see Figure 8).

(d) Repeat date with increased starting weight given the low collagen yield of the first run.

(e) VERA-4048 was assessed as suspect due to a low CO_2_ yield and, hence, repeatedly dated.

(f) Determined with EA-IRMS for gelatin extracted from a subsample of the tooth following the same chemical procedure used for the dated sample. [EA-IRMS measurement precision of a repeatedly measured standard material: 0.1‰ (1 σ) for δ^15^N and δ^13^C].

(g) Determined with the AMS system in the graphitized sample.

The first stage of the project consisted of the dating of four samples—the three anthropically modified bones plus one unmodified shaft fragment from layer 3—in the ORAU laboratory (Oxford Radiocarbon Accelerator Unit). The layer 3 sample failed, as did two similar replacement samples (Figure S5), while the results for the others, when read in light of their stratigraphic provenience, bracketed the Aurignacian occupation of the site but could not settle the issue of its exact age. It was thus decided to submit a second batch, comprised of material from *in situ* areas of layer 2 and intended to date by association the stone tool assemblage recovered therein; ten more bones were sent to Oxford (all of which eventually failed), and four teeth were sent to the VERA laboratory (Vienna Environmental Research Accelerator).

The 7th century AD result for OxA-15005 was obtained on a bone fragment of mineralized appearance (Figure 6A), and supports the interpretation of the bulk bone ICEN-490 result (ca.23 ka ^14^C BP) as rejuvenated by residual non-Pleistocene contaminants undetected at the time of sample selection. In fact, such a result would be the outcome of having 3% of that sample (i.e., 9 g, or the equivalent of a single small shaft fragment) be of the same medieval age as OxA-15005 and the remaining 97% of the same Aurignacian age as the bone fragments in the ICEN-732 sample dated to ca.28 ka ^14^C BP.

The collagen yield (5.4 mg, <1% of the initial weight) of sample OxA-15499 (Figure 6B) was below the threshold normally used at Oxford to pass samples (10 mg and 1% of the initial weight), but the carbon yield (53%C) and C∶N ratio (3.3) were within the acceptance range. A second attempt, using an increased initial weight, failed to ameliorate the collagen yield, but all other quality indicators were equally good and the result (OxA-X-2272-25) was indistinguishable. Although the replication of the initial result strengthens our confidence in its accuracy, we cannot exclude the possibility that both represent minimum ages only and that the true age of the sample is the same as that obtained for the other anthropically modified bone found in a similar stratigraphic position, at the interface between layers 2 and 3 (Figure 6C): ca.42.8 ka cal BP (OxA-15004). We therefore conservatively infer from these results a *terminus post quem* of ca.43 ka cal BP for the contents of overlying layer 2, but note that, if OxA-15499 is not just a minimum age, then the base line for the subsequent occupation of the cave is ca.40 ka cal BP.

The ambiguous stratigraphic provenience of these two samples precludes certainty as to the nature of their relationship with the fauna and lithics from layer 3. There are two possibilities: (a) the bones derive from layer 3, which they date by association; or (b) they were introduced during a sedimentation hiatus between the end of the deposition of layer 3 and the beginning of the deposition of layer 2 (the existence of such a hiatus is implied by the marked discontinuity observed at the interface between the two units in non-disturbed areas of the cave; see Figures 5, S1, S2).

At first glance, the fact that its fauna relates to lynx denning and its few flints are in secondary position makes it unlikely that layer 3 contained anthropically modified bones, implying rejection of the first possibility. However, the only area available for human shelter during the accumulation of layers 2 and 3 was that of the 1960s trench (Figure 4), and the dated samples were collected adjacent to that trench, on the entrance side of it (Figure 8); in contrast, most fauna and lithics came from squares M13-14, towards the back of the cave. Moreover, the dated bones bore no signs of having been washed in (e.g., abrasion from transport by natural agents). These anthropically modified faunal samples could therefore relate to a very ephemeral Middle Paleolithic occupation of the cave interior, the archeological remains of which were once to be found in layer 3 deposits of squares L-M12. Unfortunately, the hypothesis cannot be tested, as such remains, if they ever existed, were recovered, missed or lost in the 1960s, and are now unavailable.

Of the four tooth samples dated in Vienna, only VERA-4048 (Figure 7D) is possibly suspect, because of a low CO_2_ yield. Subsequent runs gave slightly younger results, and failure to obtain a measurement from the <30 kDa fraction means that the impact of possible contaminants cannot be assessed. Where VERA-4047 (Figure 7B) and VERA-4050 (Figure 7A) are concerned, indistinguishable results were obtained for subsamples pretreated with the standard method and with ultrafiltration. The ultrafiltrated subsample of VERA-4049 (Figure 7C) gave the same result as the subsample with standard pretreatment but was entirely <30 kDa, assumed to be the less reliable fraction; both subsamples, however, are statistically identical to VERA-4050. Taken together, therefore, this evidence shows that a faunal component with an age of ca.33.5–34.5 ka cal BP is present in layer 2.

The Vienna and Oxford sets of dates are in stratigraphic order, and, when dating other sites in the ≥30 ka ^14^C BP range, the two laboratories have in the past produced indistinguishable results for subsamples of the same bone, or for samples of different bones from the same stratigraphic context (e.g., the Oase project [28]). Moreover, the temporal hiatus between the two sets is consistent with the stratigraphic discontinuity observed at the interface between layers 2 and 3, and the existence of such hiatuses at this point in time is a generalized feature of cave and rock shelter sequences of southwest Iberia that span the Middle-to-Upper Paleolithic transition [19], [69]–[71].

None of the 14 bone samples submitted was dated to the range of the Gravettian or of the later Upper Paleolithic, and the ten from layer 2 that failed to pass the OxA quality controls provide negative evidence that no Pleistocene bone of post-Aurignacian age exists in the deposit. In part, the failure may relate to the fact that six of those ten bones were of juveniles, reflecting the composition of the faunal assemblage but meaning a less compact bone tissue, potentiating collagen leaching and explaining why the yields were too low for dating. This explanation, however, does not apply to the four failed layer 2 samples that came from adult animals (Table S3); indeed, preservation in samples of adult ibex, deer and horse from Gravettian, Solutrean and Magdalenian cave and rock shelter contexts of Portugal submitted for AMS radiocarbon dating at Oxford (14 samples from five different sites with similar geochemical conditions—Lapa do Anecrial, Buraca Escura, Buraca Grande, Gruta do Caldeirão and Abrigo do Lagar Velho) has so far proven good enough to warrant extraction of collagen within the laboratory's standards, yielding results ranging from 13,050±100 ^14^C BP (OxA-5522) to 26,020±320 ^14^C BP (OxA-5542) [54], [68], [72]–[73]. Bearing in mind that, despite significant inter- and intra-site variability, the collagen content of bones decreases as a function of burial time [74], the overall chemistry of the Pego do Diabo samples, including the failed ones, is therefore thoroughly consistent with an Aurignacian or Mousterian age for the totality of the Pleistocene faunal assemblage recovered in layers 2 and 3.

At any given site, when animal bones are accumulated by humans, they may conceivably represent only a short glimpse of activity, not the entire duration of the interval of accumulation of the deposit that contains them. In layer 2 of Pego do Diabo, however, the fauna is primarily non-anthropogenic; therefore, the age range obtained for the dated bones defines the time of formation of that layer, bracketing the age of its contents, including the stone tools, to the narrow time window indicated (in agreement with the homogeneity of the lithic assemblage) by the successful samples. Moreover, the 14 samples from the *in situ* areas of layer 2 submitted for direct AMS dating represent 42% of all piece-plotted bones with that provenience and, directly or indirectly, indicate a pre-Gravettian age. The probability that an *in situ* archeological component of Gravettian or later Upper Paleolithic age exists in layer 2 is therefore so small that we can confidently reject the hypothesis and maintain a pre-Gravettian age for the lithic assemblage recovered therein.

### The Non-Aurignacian Microlith

The only issue left unresolved by the new AMS radiocarbon results is the chronology of a microlith of REM provenience (i.e., found in the disturbed sediments from profile collapse collected from the bottom of the 1960s trench prior to the 1988–89 excavation work). It has an unpatinated surface appearance, completely different from that of the excavated lithics, and its markedly curved profile sets it apart from that assemblage on technological grounds (Figure S9).

This microlith has been used to support the case for a Gravettian age of the Pego do Diabo lithics on the basis that it would be a “small microgravette point made of red flint” [55]. However, it is neither red flint nor a microgravette. Fusiform bipoint, in agreement with overall shape and proportions, is a possible classification, but one that is inconsistent with the object's profile, so elongated segment is a better fit. In Portugal, fusiform bipoints occur as a rare type in the Upper Magdalenian [54] and elongated segments exist in the Mesolithic and Neolithic [75], but neither type has ever been found in the Gravettian [54]. We conclude that this unpatinated microlith was abandoned on the surface of the site in Tardiglacial or early/mid Holocene times, periods during which the cave remained open and accessible, and we infer for it the same original stratigraphic position—layer 1—as for the ceramics and sheep/goat teeth of same REM provenience. In short, neither typology nor stratigraphic association support any relation whatsoever between the object, the Gravettian and the human occupation in layer 2.

We further note that human osteological remains were found in layers A and 1, or at their interface with underlying layer 2, in all of the squares surrounding the 1960s trench (J12, L-M11, M13-14) (Table S4). This spatial context raises the possibility that the microlith entered the cave in the framework of Neolithic funerary activities, ones whose more conspicuous skeletal remains would have been taken out by the 1960s excavators. The deposition of bodies accompanied by stone tools (blades, microliths, polished axes) and ornaments but no ceramics was common practice in the Late Neolithic of the region, as documented by sealed cave sites containing hundreds of skeletons (e.g., Bomsanto and Lugar do Canto) [75]. In the immediate vicinity, Neolithic funerary use of such small caves as Pego do Diabo is attested by the site of Gruta dos Penedos, <1 km away [76], and according to Carl Harpsøe (personal communication, March 07, 2009), a group from the Palethnology Department of the Portuguese Speleological Society recovered a polished adze in a 1965 or 1966 visit to Pego do Diabo.

In order to test the hypothesis that this adze, the unpatinated segment and the human osteological remains did define a Late Neolithic burial context, we dated two human bone samples recovered during the 1988–89 excavations: J12-2, a juvenile scapula from layer 1; and J12sc24, a hand phalanx found in the same disturbed area against the wall of the cave that extends down to the interface between layers 2 and 3 (Figure 8) whence also came the OxA-15004 Mousterian sample discussed above. The results obtained (Table S5) are fully consistent with the hypothesis, as they prove burial use of the cave ca.3000 cal BC, i.e., the time range indicated by dates for similar contexts elsewhere in Portugal.

### The Dufour Bladelets

Bearing in mind the palimpsest nature of cave deposits, the dating of layer 2 to the time range of the Aurignacian III–IV does not completely reject the possibility that the artifacts contained therein entered the site at some point in time during the hiatus between the deposition of layers 2 and 3, i.e., in the ca.35–43 ka cal BP interval. Confirmation that the Pego do Diabo Dufour bladelets are indeed Aurignacian III–IV therefore requires assessment of whether their metrical and formal attributes are consistent with alternative assignments to earlier stages of the technocomplex.

A persistent source of confusion in the study of the Aurignacian is the vague, catch-all original definition of the “Dufour bladelet” type: “bladelet with a curved profile, presenting a fine, marginal, semi-abrupt retouch, along one of the edges only (in which case it can be either ventral or dorsal) or along both edges (in which case it is always alternate)” [57]. As a result, over the years, practitioners have subsumed under this category an extremely varied range of microliths with very little in common in terms of blank technology, mode of retouch, and overall shape.

A case in point is the putative presence of Dufour bladelets in Châtelperronian level X of the Grotte du Renne, at Arcy-sur-Cure [77], which some have used to support the twin notions that the site is heavily disturbed and that the numerous ornaments found in level X originated in Aurignacian level VII, where Dufour bladelets are abundant [78]–[79]. In fact, the few level X items in question represent one end of the variation of the “retouched blade” tool type. They are not bladelets but blades (their average width is 13.5 mm), and they display a technology of blank production that is distinctively Châtelperronian [80].

**Figure 9:**
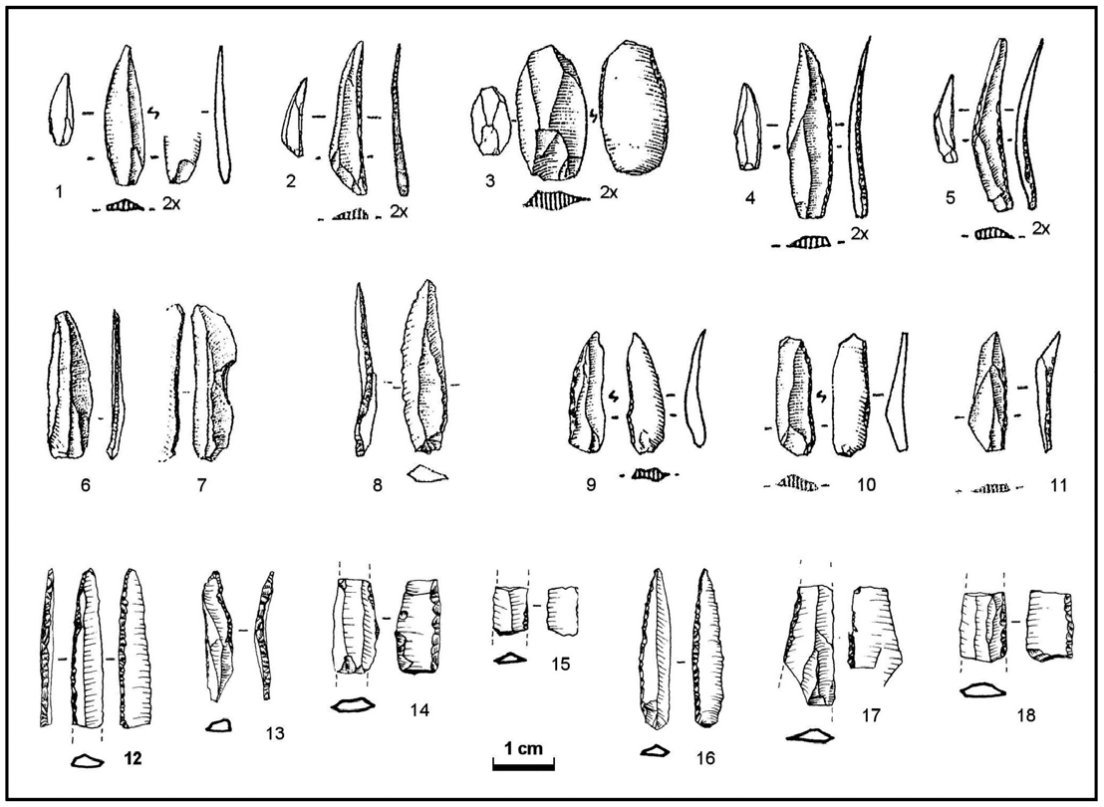
“Dufour bladelets”: in the Portuguese Upper Paleolithic [54]. 1–5, 9–11. Early Magdalenian of Cerrado Novo; **6–8.** Gravettian of Vale Comprido – Barraca and Terra do Manuel; **12–15.** Pego do Diabo layer 2 (intact Pleistocene deposits); **16–18.** Pego do Diabo layer 2D (Pleistocene deposits with Holocene intrusions). These items all fit the rather vague consensus definition of the Dufour bladelet type, although no. 13 is classified separately in Table 2 because it features bilateral direct retouch. The non-Aurignacian Dufours differ from the Pego do Diabo material in size, mode of retouch and blank technology (see Table 8). Note, in particular, the hypermicrolithic nature of the Early Magdalenian specimens, and the obtuse (or naturally pointed) distal extremities and marginal retouch of the Gravettian ones. In contrast, the material from Pego do Diabo is elongated, more robust, bilaterally pointed, and features ventral sides modified via semi-abrupt, often invasive retouch of a kind never used in the Gravettian or the Magdalenian.

The argument concerning the existence of Dufour bladelets in the Gravettian of Portugal is of a similar nature (Figure 9; Table 8). Bladelets with an inverse retouch occur in such assemblages (as well as in Magdalenian and even Early Mesolithic ones), but (a) they are always a small proportion of the bladelet tool category (Table S6), (b) the retouch is almost always unilateral and very marginal, never the kind of alternate, semi-abrupt, invasive retouch seen in the Pego do Diabo material, and (c) in terms of module, they are poorly standardized (reflecting the type's essential heterogeneity), contrasting with the tighter control over width and thickness (a reflection of the underlying, specific technology of blank production) seen in the Pego do Diabo Dufours (Figure S10).

**Table 8:** Pego do Diabo vs. the Portuguese Gravettian and the Protoaurignacian of Grotte du Renne level VII: attributes and metrics of the Dufour bladelet category (a).

Assemblage	N	Width (b)	Thickness	Carination Index (c)	Mounting on the shaft	Profile of the blank (d)	Retouch is alternate, not inverse or direct (e)	Inverse retouch is semi-abrupt (f)	Inverse retouch is on the right (g)	Tip is pointed by retouch (h)
PGD 2+2D	7	6.0±1.4	1.8±0.2	3.3±0.6	Axial	Straight	86%	100%	50%	100%
Gravettian	26	7.2±1.6	1.9±0.5	3.9±1.0	Lateral	Curved	19%	0%	17%	0%
Renne VII	283	7.3±1.8	2.0±1.7	3.8±1.0	Lateral	Curved	8%	22%	97%	17%

(a) Measurements are in mm. Dufour bladelets are defined inclusively, as in the type-list [57], i.e., subsuming bladelets with only direct, marginal retouch. Grotte du Renne data for retouch mode, inverse-retouched side, and tool tip, are from [77], while metrics and extent of retouch are from [89]; data for the Portuguese Gravettian are from the sites in Table S6 [54].

(b) The original width of the blank prior to retouch could be estimated for 78% of the Grotte du Renne material (N = 221): 8.1±2.0 mm.

(c) Width/thickness.

(d) For the Grotte du Renne complete pieces (N = 10), the height of the arc defined by the ventral side when lying on a flat surface is 1.6±0.9 mm, and the curvature index (length/height) is 5.2±2.3; for the Pego do Diabo material, blank profile is straight in the six Dufour bladelets with alternate retouch (Fig. 9, nos. 12, 14–18; Fig. 11B–D), but curved in the one piece with bilateral direct, marginal retouch (Fig. 9, no. 13; Fig. 11E).

(e) The retouch of the remainder is: inverse (92%) for the Grotte du Renne; bilateral-direct and marginal (14%) for Pego do Diabo; bilateral-inverse (4%), inverse (46%), or direct and marginal (31%), for the Gravettian assemblages.

(f) At the Grotte du Renne, the inverse retouch of the other 78% is marginal (60%) or abrupt marginal (18%).

(g) For the Portuguese Gravettian, based on the 12 pieces with unilateral inverse retouch (the attribute was not recorded in the alternate-retouched specimens from this period); for the Grotte du Renne and Pego do Diabo, based on all pieces with either unilateral-inverse or alternate retouch.

(h) As opposed to obtuse or naturally pointed; percentage values calculated over the number of whole or sufficiently complete specimens (at Pego do Diabo, two entire and one distal, plus two mesial fragments—Fig. 9, nos. 14, 17; Fig. 11C—whose distally converging morphology implies a retouch-pointed tip).

**Figure 10:**
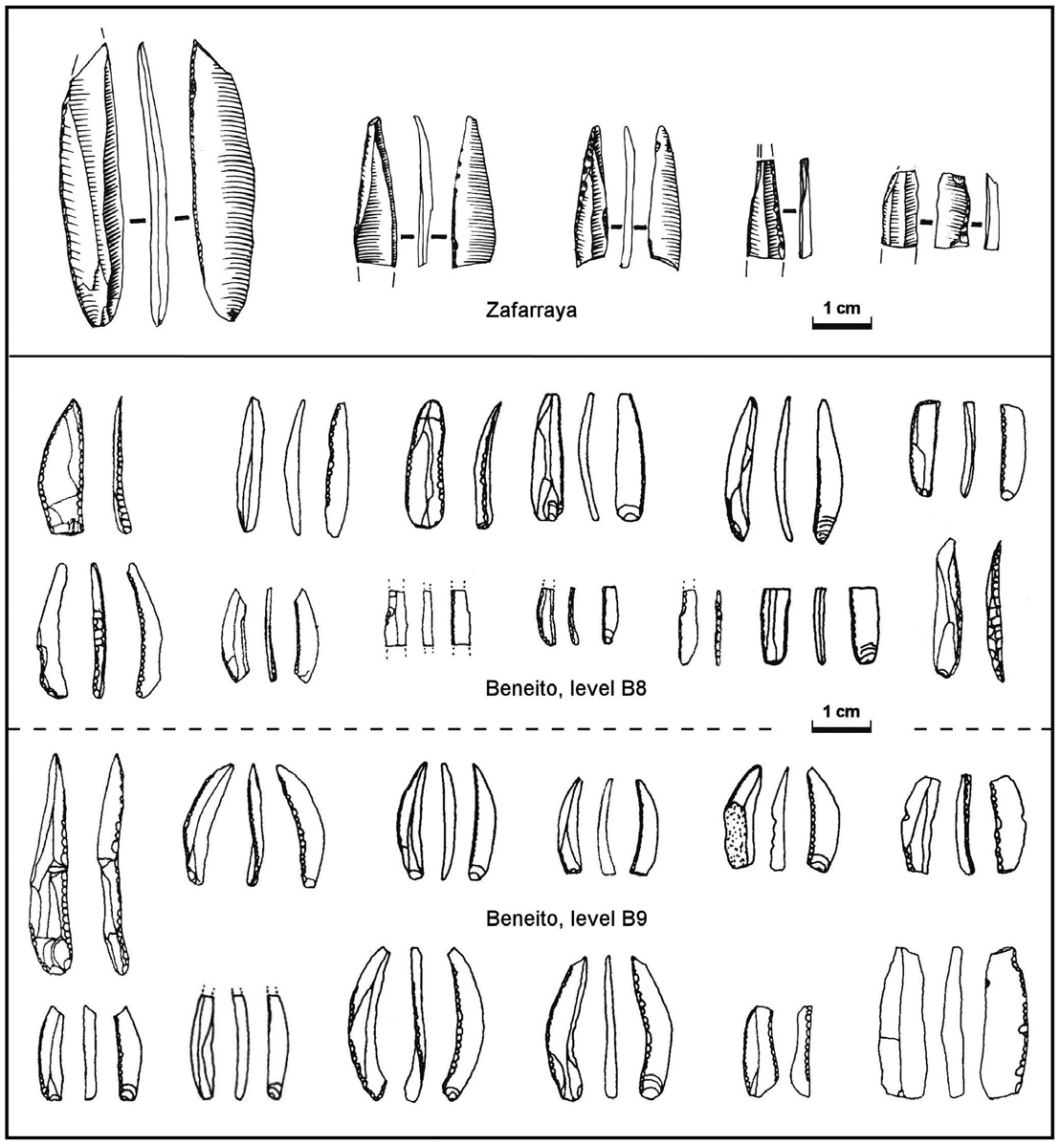
“Dufour bladelets”: in the Early Upper Paleolithic of southern Spain. With the exception of the longer piece from level B9, the material from Cova Beneito [86] fits the definition of the Roc-de-Combe subtype, while the Zafarraya pieces [87] are identical to those from Pego do Diabo.

Recent developments in the study of how technology and typology changed over time in the Aurignacian have brought to light a consistent pattern of successive Dufour bladelet subtypes [81]–[85]. In the Protoaurignacian, the blanks, extracted from prismatic cores, are elongated and curved, and a large proportion of these bladelets is modified by inverse retouch. In the Aurignacian I, the blanks remain curved in profile but are smaller (because they are now extracted from a specialized type of core, the so-called carinated “scraper”), and very rarely retouched (and, when this is the case, they bear a very fine marginal retouch, either ventral or dorsal). A change from carinated to nosed “scrapers” (plus the addition of busked “burins” to the repertoire of specialized bladelet cores) characterizes the Aurignacian II, and, in typology, generates the highly standardized Roc-de-Combe subtype [58]—made on a very small (<20 mm long), distally twisted blank, and bearing a fine, marginal retouch, almost always inverse (Figure 10).

**Figure 11:**
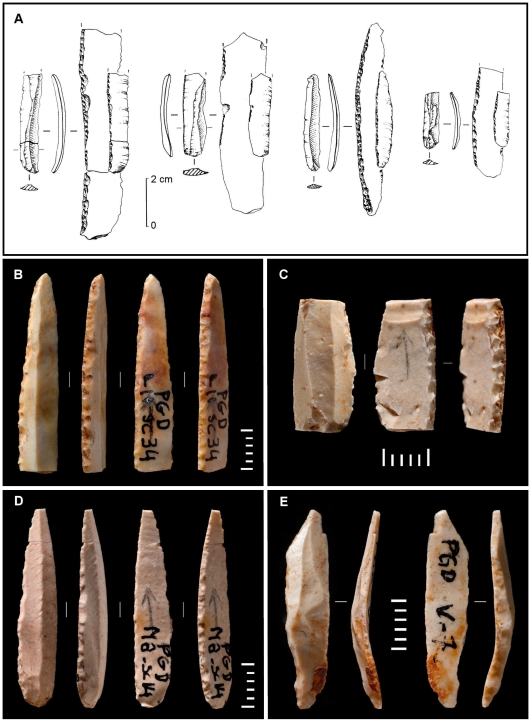
“Dufour bladelets”: Pego do Diabo compared with the Protoaurignacian. **A.** Dufour bladelets from level VII of the Grotte du Renne, Arcy-sur-Cure (France) [77]; the ventral side is shown at 2×. **B–E.** Dufour bladelets from the Aurignacian III–IV of Pego do Diabo (all scales are in mm). The straight profiles, alternate retouch, pointed tips and impact fractures of the Pego do Diabo items set them apart from the Protoaurignacian Dufours, which feature curved profiles, marginal inverse retouch and obtuse tips.

From the above (a) the Pego do Diabo Dufour bladelets are not of the Roc-de-Combe subtype, and (b) their blanks do not fit the production technology of the Aurignacian I. In order to assess the possibility that they embody the existence of a true Protoaurignacian in Portugal, we compared them with the only assemblage of Protoaurignacian Dufour bladelets that is both stratigraphically coherent and studied with the necessary level of detail—that from level VII of the Grotte du Renne [77], [88]–[89] (Table 8). Despite the imbalance in corpus size, the black/white contrasts mean that the differences are significant: in the Renne VII assemblage (Figure 11A), blanks are curved, the retouch is almost always inverse, highly lateralized, and spares the distal extremity, which remains obtuse; at Pego do Diabo, blanks are narrower, more standardized in width and completely straight, the retouch is always alternate, and it converges at the tip to form a sharp point (e.g., Figures 11B, 11D). Moreover, use-wear suggests that the Renne VII Dufours were mounted laterally, as barbs or cutting edges [77], whereas the impact fractures apparent in mesial fragment M11sc67 of Pego do Diabo (Figure 11C) implicate use as a point mounted axially on the shaft of a composite projectile.

Because of the ephemeral, logistical nature of the site's occupation, there was no debitage debris at Pego do Diabo that could provide direct information on lithic production. However, the evidence from contemporary workshop sites of the later Aurignacian of the region, such as Vale de Porcos, suggests that the Pego do Diabo Dufour bladelets were part of a system whereby the blanks intended for transformation into microlithic points were extracted from cores of the carinated “burin” type (including so-called Vachons “burins”) [18], [41], [54], [90]. This technology enables the generation of long, straight and robust blanks through the use of the edges of thick flakes as the guiding ridges of the reduction process, a method that also allows for a tight control of blank width. Such blanks, however, do not embody an exclusive intention, and the technology is so flexible that a wide range of products can be obtained [85]. A case in point is the other microlith from layer 2 (Figure 11E). It was stratigraphically *in situ* and has the same patina as the rest of the Dufours—despite the bilateral-direct retouch, there is no doubt that it belongs in the assemblage. Its bidirectional dorsal scar pattern is consistent with extraction from a carinated “burin” and excludes extraction from a carinated or a nosed “scraper;” on the other hand, it is too small, too curved and too thin to have come from the exploitation of the debitage face of a prismatic core.

This technological system is characteristic of the Aurignacian III–IV as defined in the classical French sequences of Abri du Facteur, Abri Pataud and La Ferrassie [85]. Because, at Facteur and La Ferrassie, the corresponding levels are poor and suffer from problems of stratigraphic integrity [91], only level VI of Pataud [92] can be compared with the Portuguese sites [41]. The conclusions of a recent technological analysis of the blade/bladelet production system documented in that level [85] replicate point by point those arrived at in the study of Vale de Porcos. The few bladelet tools, however, differ from those of Pego do Diabo in that, except for a couple of inversely retouched Dufour bladelets, they are all of the Font-Yves type, i.e., straight and pointed, as at Pego do Diabo, but modified by bilateral direct retouch instead of alternate retouch.

Given the scarcity of the data, the significance of this difference is unclear. Alternate retouch predominates among the small set of pointed Dufours recovered in disturbed deposits at the top of the Mousterian sequence of Zafarraya cave (Malaga, Spain) [87] (Figure 10). Combined with their typology, this stratigraphic position suggests that this material is an Andalucian manifestation of the same cultural phenomenon documented in layer 2 of Pego do Diabo [41], and the same has been suggested for identical material recovered in mixed Early Upper Paleolithic contexts at the cave sites of Salemas and Escoural, in Portugal [41], [54]. It is possible, therefore, that the details of blank retouch whereby Pataud 6 differs from the Iberian sites are a reflection of cultural preferences and, as such, simply highlight that some level of regional variation existed in the latest Aurignacian.

The issue is further complicated by the fact that the bladelet assemblage from the eponymous site of the Font-Yves point (the Grotte de Font-Yves, Corrèze, France) seems to be the byproduct of significant stratigraphic admixture, with recent re-analysis showing the representation of components of bladelet production systems characteristic not only of the Aurignacian I, II and III–IV but also of the Gravettian [85]. Moreover, level VI of Pataud is directly overlain by Early Gravettian deposits, and some disturbance existed at the interface, as further suggested by the AMS date of 26,600±800 ^14^C BP (OxA-689) obtained on a bone from that level [92]–[93]. If Gravettian intrusions are indeed present in these contexts, we must then consider the hypothesis that the Font-Yves points are early Gravettian instead of later Aurignacian.

A third possibility is that the Aurignacian III–IV featured a wide range of functionally specialized bladelet tools that, while sharing a similar, flexible technology of blank production, differed in mode of retouch. Under this model, the contrast between the Iberian sites and Pataud level 6 could be put down to site function. The fact that items with a bilateral-direct retouch exist in Dufour bladelet assemblages not only at Pego do Diabo but also at Zafarraya is consistent with the hypothesis.

Where the dating of the later Aurignacian is concerned, we must bear in mind that the anomalously young result for Pataud 6, obtained by AMS but in the initial stages of the method, can also reflect incomplete sample decontamination. This is almost certainly the explanation for the discrepancies in the corpus of dates for the latest Aurignacian levels of La Ferrassie, mostly made up of conventional results obtained on bulk bone samples. However, the single AMS date available for this cultural horizon—OxA-405 (29,000±850 ^14^C BP), obtained on a bone from level G1 of the sagittal profile [94]—overlaps with the time range of Pego do Diabo layer 2. This is also the case with a conventional date obtained for Pataud 6 on a sample of unknown composition: 28,510±280 ^14^C BP (GrN-6273) [92]. Thus, although scant, the chronometric information available for the few stratified Aurignacian III–IV contexts of France is in agreement with regional chronostratigraphic patterns, as it places them in the interval of time between the Aurignacian II and the Early Gravettian.

## Discussion

The bladelet tools from layer 2 of Pego do Diabo are associated with a faunal assemblage of Early Upper Paleolithic composition and dated by chemically reliable samples to the time range of the Aurignacian III–IV. Besides scant recent Holocene intrusions, no other component exists in these deposits, as corroborated by the fact that, despite having consumed in the process 42% of all the piece-plotted bones recorded therein, our dating project returned for samples from layers 2 and 2D no results of intermediate age. Typological and technological considerations exclude assignment of the Pego do Diabo Dufours to earlier phases of the Aurignacian or to the Gravettian. The inescapable conclusion is that a human occupation of Aurignacian III–IV cultural affinities took place at Pego do Diabo ca.29–30 ka ^14^C BP (ca.33.5–34.5 ka cal BP), i.e., in the lower end of the 95% confidence interval of the conventional bulk bone date obtained 20 years ago.

The Aurignacian-to-Gravettian transition is one of the least known periods of European prehistory, explaining why the evidence from such a small site as Pego do Diabo can contribute to our understanding of this process. In particular, our results put the west European evidence in line with that from central Europe, where the earliest Gravettian is now dated to the ca.29–30 ka ^14^C BP interval [95]. This transition was therefore penecontemporaneous at the continental scale: the south German pattern is no instance of precocity, as in the *Kulturpumpe* model [95], nor is it necessarily a byproduct of post-depositional displacement of samples derived from underlying Aurignacian levels, as others have proposed [30].

Our results also highlight the need to improve the definition of the Dufour bladelet type. It has already been suggested [85] that the term “Font-Yves” should not be used as a synonym for the rather different El Wad/Krems points that often occur alongside Dufour bladelets in Protoaurignacian contexts of eastern, central and western Europe [35], [83], [96], but the different types currently subsumed in the “Dufour bladelet” umbrella category that have a restricted space-time distribution must also be extracted from it. The axially mounted micropoints obtained by the alternate retouch of straight elongated blanks using semi-abrupt, somewhat invasive retouch of the ventral side, and direct, continuous retouch of the opposite dorsal side, are probably carriers of such a kind of cultural or chronostratigraphic information. If future research confirms that they are indeed exclusive of the Aurignacian III–IV of at least Iberia, then a new type should be formally created out of this definition: the “Pego do Diabo point.”

**Figure 12:**
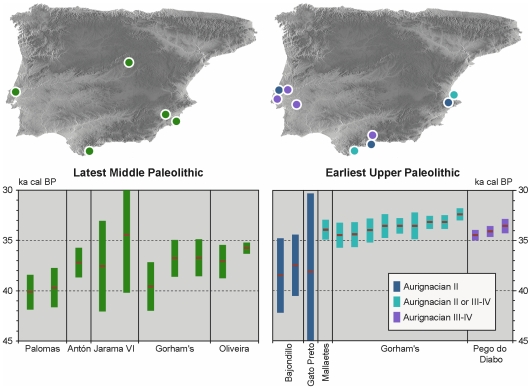
Geography and chronology of the Middle-to-Upper Paleolithic transition in southwest Iberia. Top, site locations, from West to East (Latest Middle Paleolithic: Gruta da Oliveira; Gorham's Cave; Jarama VI; Cueva Antón; Sima de las Palomas. Earliest Upper Paleolithic: Pego do Diabo and Gruta de Salemas; Gato Preto; Vale de Porcos; Gruta do Escoural; Gorham's Cave; Cueva Bajondillo; Cueva de Zafarraya; Cueva Beneito; Cueva de Mallaetes). Bottom, plot of the mid-points and 95% confidence intervals of the AMS radiocarbon results for layer 2 of Pego do Diabo compared with the dataset in Table S7 (only sites and dates that passed taphonomic critique are retained [19], [50]; the age for Gato Preto derives from the TL dating of burnt flint; the ages for the other sites derive from calibrated radiocarbon results on charcoal and bone).

Where the emergence of anatomical modernity is concerned, Pego do Diabo establishes a secure *terminus ante quem* of ca.34.5 ka cal BP for the process in central Portugal, where, on current evidence [19], [39], [41], the *terminus ante quem* for the demise of Neandertals is ca.35.5 ka cal BP (Table S7; Figure 12). This has major implications for the interpretation of the archaic features in the anatomy of the Lagar Velho child. With the last of the region's Neandertals dating to five millennia before the child was borne, crossbreeding between immediate ancestors (e.g., parents or grandparents) drawn from distinct “modern” and “Neandertal” gene pools is empirically untenable. Therefore, those features must represent evolutionarily significant admixture at the time of contact.

A second implication of the Pego do Diabo results is that they enable us to constrain the uncertainty of the (TL) Thermoluminescence date for the open air site of Gato Preto (Rio Maior), in the same region. The date is consistent with the Aurignacian II affinities of the lithic assemblage, and with the chronological anteriority to Pego do Diabo implied by those affinities. Moreover, an Aurignacian II also exists in southern Spain, namely at Cova Beneito (Alicante, Valencia; levels B8-B9; Figure 10) and Cueva Bajondillo (Malaga, Andalucía; level 11). The latter is dated with significant imprecision, further impaired by issues of sample quality and association [19]. However, the results indicate an age interval consistent with the evidence from Pego do Diabo and Gato Preto (Figure 12), as well as with the chronology of the Aurignacian II across Europe, where reliably dated occurrences (those dated on charcoal samples from hearth features or by AMS on bone [29]), such as Pataud level 8 [93], or the Austrian open air sites of Willendorf II and Stratzing [97]–[98], fall in the ca.30–33 ka ^14^C BP interval (ca.34–37 ka cal BP).

One can thus surmise for the Aurignacian II of Iberia a chronology centered around 36.0 ka cal BP, and a geographic distribution encompassing at least the entire length of the peninsula's littoral. The radiocarbon results for the Middle Paleolithic levels of the Jarama VI cave, in Guadalajara, are consistent with this picture, although, given their large standard deviations, they leave open the possibility that Neandertals persisted in central Spain for a couple of millennia longer. At first glance, the hypothesis is comforted by the fact that, in the interior Meseta plateaux, the Aurignacian remains unknown. In these regions, however, it is the entire Upper Paleolithic, not just the Aurignacian, that is absent or very poorly documented, due to preservation and research biases. Therefore, the parsimonious reading of the Jarama VI dates is that they do not question the pattern seen in the littoral areas.

In firmly anchoring the chronology of the later Aurignacian of southwest Iberia, the Pego do Diabo evidence also has implications for the notion that Neandertal populations remained in the Gibraltar area until ca.28–32 ka cal BP [47]–[48]. As hunter-gatherers living at low population densities, residual Neandertals could not have survived for several millennia as a separate biological entity unless they controlled a territory large enough to sustain a bounded, viable reproductive network of at least several hundred people, i.e., a territory in the range of the tens of thousands of square kilometers. Thus, in and by itself, the Aurignacian II evidence from Malaga, 150 km to the Northeast, would not be inconsistent with a very late Neandertal settlement of the Gibraltar area provided that such a settlement represented the eastern frontier of a refugium that extended all the way to the western façade of Iberia. But the data from Pego do Diabo and the other Portuguese Aurignacian sites show that such was not the case.

Given that no secure evidence exists elsewhere for peninsular Neandertals to have persisted beyond the 37th millennium cal BP, and that available archeological proxies place the emergence of anatomical modernity across southwest Iberia no later than the beginning of the 36th, the younger dates obtained for the Middle Paleolithic of Gorham's Cave must be considered anomalous [50]. These dates are part of a large set of results, mostly within the expected range but widely scattered and with no correlation between age and stratigraphic depth. Combined with the microscopic size of the charcoal samples used, this pattern means that incomplete decontamination and post-depositional intrusion from the overlying Upper Paleolithic are viable explanations for the outliers. Moreover, the samples come from a trench in the back part of the site where find densities are very low (five artifacts per cubic meter) and a non-diagnostic Upper Paleolithic stone tool component may well exist alongside the few clearly Middle Paleolithic items. Finally, Upper Paleolithic deposits of later Aurignacian affinities exist in the porch area of the site and are well dated by numerous samples to ca.34 ka cal BP (Table S7; Figure 12). This dating is in line with the evidence from Pego do Diabo, and precludes the possibility that the back area of Gorham's continued to be used by Middle Paleolithic Neandertals beyond that point in time.

Why, south of the Ebro drainage system, the replacement/assimilation process occurred much later than elsewhere in Western Europe, remains a “big issue.” Our hypothesis is that climatic and demographic factors are involved [13], [21]–[22]. North of the Pyrenees, the impact of a severely cold iceberg event (Heinrich Event 4), aggravated, in central and eastern Europe, by the effects of the Phlegraean Fields caldera explosion, must have caused a population crash. At the same time, to the south, and especially so along the Atlantic façade, oak and pine woodlands expanded significantly during the period of the “Ebro Frontier,” which, globally, was one of generally milder climate (GIS8; Greenland Interstadial 8); by comparison with what happened in Iberia at the time of the Tardiglacial/Early Holocene transition, population decrease and a break-up of interaction networks probably occurred as a result of the expansion of such tree-covered landscapes. The net result may have been one where, for modern human groups settling the foothills of the Cantabro-Pyrenean mountains, southward expansion (or networking) may have become neither possible nor desirable. Then, as population numbers recovered and the long GIS8 interval came to an end, with southwest Iberian environments opening up for large herbivore herds and their hunters as a result of the return to stadial conditions, interaction and movement across the previous boundary must have ensued, with inevitable consequences for the Neandertal refugia of westernmost Eurasia.

## Materials and Methods

All animal bones and teeth were examined, but only certain regions of some of the bones were recorded in detail and counted. The criteria applied when deciding whether to record a particular fragment of bone or tooth, and how they are counted, are described elsewhere [65], [99]. The Parts of the Skeleton Always Counted (PoSAC) are similar to Watson's “diagnostic zones” [100]. For example, the medial half of the articulation of the distal tibia is counted, but none of the following parts of a tibia would be counted: the lateral half of the distal articulation, diaphysis, and proximal end. These “counted parts of the skeleton” include the mandibular cheek teeth, and articular ends/epiphyses and metaphyses of girdle, limb and feet bones. They are the units used to calculate the frequencies of different parts of the skeleton and proportions of young (epiphysis unfused) versus adult (epiphysis fused) animals. When other parts of the skeleton such as antlers, horn cores or maxillary teeth are the only evidence for the presence of a species, these non countable specimens are recorded and their presence denoted by a “+” sign, but not included in the total counts of species found. The reasons for selecting these particular parts are as follows: a) they are relatively easy to identify to species; b) some, such as the distal metacarpal in certain species of artiodactyls, when in sufficient quantity, can provide information about the sex ratio; c) many include a separate centre of ossification, or epiphysis, which fuses to the rest of the bone at a particular age and so, in sufficient quantity, provide a ratio of juveniles to adults; d) many provide useful measurements; and e) they come from most regions of the skeleton (head, girdles, limbs and feet) and their relative abundance indicates possible preferences for different parts of the body such as non-meat bearing versus meat bearing parts or fore quarters versus hind quarters.

At the ICEN lab, samples were first cleaned by hand removal of foreign material. For bone samples, gelatin was extracted using the Longin method [101], and the ^14^C measured by means of the liquid scintillation technique described elsewhere [102]. Stable isotope enrichment values (δ^13^C) were determined for the CO_2_ gas produced in the initial stage of benzene synthesis. Radiocarbon ages were calculated in accordance with the recommendations of Stuiver and Polach [103]. The gelatin extracted from ICEN-490 and ICEN-732 was pure, with no residue after combustion, and normal δ^13^C values were obtained, whereas the gelatin of ICEN-491 was impure, the yield very low, and the δ^13^C could not be measured due to an accident in the mass spectrometer (Table 3). For these reasons, ICEN-490 and ICEN-732 are reliable measurements, but ICEN-491 is not.

Extraction of collagen at ORAU followed the ultrafiltration protocol [63]. Gelatin was extracted and purified using an acid-base-acid treatment, followed by gelatinization and filtration to remove large insoluble contaminants. Subsequently, the >30 kDa fraction from a pre-cleaned VivaspinTM15 ultrafilter was collected removing the smallest contaminants and degraded collagen. For a reliable date, 10 mg of collagen should be obtained representing a >1% yield. Samples were combusted in a Roboprep CHN sample converter unit, operating in continuous flow mode interfaced with a Europa Scientific ANCA-MS system consisting of a 20-20 IR mass spectrometer allowing measurement of δ^13^C, %C and C∶N ratio. Acceptance values are -22- to -18‰, >30%, and 2.9–3.4 respectively [74]. On the basis of these values, OxA-15004 and 15005 are reliable, whilst OxA-15499 and its repeat OxA-X-2272-25 may be less so because of their low % and mg yields; they should thus be regarded as minimum ages. Samples were graphitized and measured in an AMS as described elsewhere [104]–[105].

A comparison of two different chemical pretreatment methods was performed at VERA for the four animal teeth from layer 2. For bones, the collagen extraction yield is widely used as a measure of the suitability of a sample for reliable ^14^C dating (see above). However, the exact yield of collagen extracted from the tooth dentin is difficult to determine, due to the possible presence in the sample of an unknown amount of enamel, which is very low in organic material. Apart from the collagen yield, some laboratories use additional parameters to characterize the quality of the extracted bone collagen, e.g., δ^13^C, δ^15^N, C/N ratio, %C, etc. [74]. These parameters are not sensitive enough to detect minimal sample contaminations which could—depending on the deviation of their ^14^C/^12^C ratio from that of the sample—lead to a severe distortion of the ^14^C dating result. Therefore, in order to have a quality indicator available for the ^14^C data of the animal teeth, different collagen extraction methods which are assumed to have a different efficacy to remove any contamination from the dated material have been applied to two subsamples of the individual tooth samples. An agreement of the ^14^C dates obtained with these methods implies that both methods were efficient to remove the contamination from the samples, or that a contamination which would affect the determined ^14^C ages was not present in the samples.

One of the applied methods was a modified version of the Longin method [101], with a base and an acid step added before gelatinization. This procedure is used as a standard method for the sample preparation of bones at VERA. The reliability of this method was recently proven once again by the VIRI (Fifth International Radiocarbon Intercomparison) bone dating exercise, in which VERA participated. The second chemical method used for the tooth samples was the ultrafiltration method, which is in essence the procedure described in [63].

Of the four dated teeth, the results for samples VERA-4050 and VERA-4047 (see Table 7) can be assessed as reliable. In both cases, the collagen yields of the standard gelatin production method are close to the 1% limit for bones. As mentioned above, these yields should be treated as minimum values due to the unknown amount of enamel present in the sample. For both samples, the ^14^C age of the >30 kDa gelatin fraction (indicated by the extension UF1 in the VERA-numbers) is in excellent agreement with the ^14^C age derived with the standard method. However, it must be noted that whereas the collagen yield of the ultrafiltered sample VERA-4047 shows a reasonable value (0.5% of the initial sample amount in both molecular weight fractions), the collagen yield of the ultrafiltered subsample of VERA-4050 is rather low. On the other hand, the ^14^C result of the <30 kDa fraction (extension UF2) from both samples only slightly deviates from the >30 kDa fraction (age differences: 1470±410 ^14^C years (1σ) for VERA-4050 and 790±380 ^14^C years (1σ) for VERA-4047), showing that no severe contamination was present in the collagen solution before the ultrafiltration. This information supports the assessment that the samples' ages are reliable.

The ^14^C data determined for the two other teeth samples must be treated with some caution. In one case (VERA-4049), the entire collagen in the ultrafiltered control subsample went into the <30 kDa fraction, which is assumed to give less reliable dates, and the collagen yields of both methods are low. Sample VERA-4048 is also problematic. In a first run of the sample, pretreated with the standard procedure, the CO_2_ pressure detected in the graphitization reactor was lower than estimated for the used collagen amount. The ^14^C results of two independent dating reruns deviate more than the expected measurement uncertainty. Although the VERA-4048UF1 age fits the two older “standard method” results, the entire set of data of this sample means that its results have to be assessed as less secure.

For all ^14^C dated samples, the mass-dependent isotope fractionation correction was derived from the ^13^C/^12^C ratios of the samples determined with the AMS system. From these ratios, the δ^13^C values of the graphitized samples and their uncertainties were calculated and are given in Tables 7 and S5 as well. Although the uncertainty of these δ^13^C values is rather high compared to the precision achieved with a stable isotope mass spectrometer, it does not affect the precision of the fractionation-corrected ^14^C age. Moreover, in ^14^C dating, it is advantageous to use the ^13^C/^12^C ratio of the graphitized sample measured with AMS because isotope fractionation introduced in the laboratory is also included in the correction.

As mentioned above, some laboratories also use C/N ratio, δ^13^C, δ^15^N and %C values determined in gelatin extracted from bone samples to characterize the dated material. At VERA, these parameters were determined in gelatin extracted with the standard method from subsamples of the teeth. The measurements were performed with an elemental analyzer coupled to a stable isotope ratio mass spectrometer (EA-IRMS system, at VERA: CE Instruments NC 2500 elemental analyzer coupled to a Micromass Optima mass spectrometer), operated in the continuous flow mode. For each of these samples the determined values fulfill the criteria for gelatin assessed to yield reliable ^14^C dating. The data also demonstrate that the δ^13^C values derived by AMS measurement agree within uncertainty with those determined with EA-IRMS.

Two human bone samples were also dated at the VERA laboratory (Table S5). A comparison of the two pretreatment methods applied in this study was performed for sample J12sc24 (VERA-4982), and the resulting ^14^C ages are in excellent agreement. Due to the better preservation (∼2% yield) of these “young” bones, enough gelatin was available after the standard pretreatment to determine the parameters for characterization of the dated gelatin by EA-IRMS directly in portions split off from the dated material. As expected, the determined additional parameters meet the criteria for reliable bone dates.

## Supporting Information

Table S1 Pego do Diabo: rabbit material in layer 3 radiocarbon sample ICEN-491 (a).
(0.10 MB PDF)

Table S2 Pego do Diabo vs. Gruta do Caldeirão: species percentages (a).
(0.12 MB PDF)

Table S3 Pego do Diabo: OxA-failed AMS samples.
(0.14 MB PDF)

Table S4 Pego do Diabo human skeletal remains.
(0.13 MB PDF)

Table S5 Pego do Diabo: AMS radiocarbon results obtained for human skeletal remains (a).
(0.12 MB PDF)

Table S6 Pego do Diabo vs. Portuguese Gravettian: relative frequencies of bladelet tool categories (a).
(0.12 MB PDF)

Table S7 Taphonomically reliable ^14^C and TL results for the Middle-to-Upper Paleolithic transition in Iberia (a).
(0.16 MB PDF)

Figure S1 Pego do Diabo: M>N14W-12 profile. Facsimile reproduction of the field drawing, with indication of the position of the profiles whose photographs are given in Figure 5. Note the post-excavation correction of the boundary between layers 2 and 3. This rectification has no implication for the assignment of finds from the 1988–89 field work, as it affects an area where the profile results from the excavation of the 1960s trench.
(9.37 MB TIF)

Figure S2 Pego do Diabo: M13>14 profile. Facsimile reproduction of the field drawing.
(9.71 MB TIF)

Figure S3 Pego do Diabo: the surface of layer 3 in squares L-M11. Facsimile reproduction of the field drawing.
(9.05 MB TIF)

Figure S4 Pego do Diabo: lithics from layers 3–4. Top: flake M13sc29, from layer 3. Bottom: flake from layer 4, square M9. Note the patina and edge damage, which suggest that the accumulation of these and the few other flints of similar appearance recovered in layers 3–4 relates to natural inwash processes, not to human activity at the site. In 1965 or 1966, a group from the Palethnology Department of the Portuguese Speleological Society collected flint flakes in ploughed fields immediately above the limestone ridge where the cave opens (Carl Harpsøe, personal communication, March 07, 2009), and such may well be the provenience of the lithics found in these layers.
(3.05 MB TIF)

Figure S5 Pego do Diabo: failed radiocarbon samples from layer 3. All come from square L11 (see Table S3 for further details). Note the manganese staining.
(2.27 MB TIF)

Figure S6 Pego do Diabo: taxonomic diversity in context. A plot of the number of mammal species (of size equal to or greater than a rabbit) against the decimal logarithm of the number of bones of mammals (of size equal to or greater than a rabbit) identified to species level from 107 archeological sites/levels in Europe and the Near East studied with the PoSAC method [65], [98]. Sheep and goat are treated as a single taxon as are the various species of equids. The numbers of bones range from 5 to 9673 and the numbers of species range from 1 to 15. The abundance of taxa observed in layer 2 of Pego do Diabo is all the more striking because of the small size of the mammal bone assemblage.
(1.44 MB TIF)

Figure S7 Pego do Diabo: OxA-failed radiocarbon samples from layer 2 (spit 2a). For taxonomic and skeletal part details, see Table S3.
(4.31 MB TIF)

Figure S8 Pego do Diabo: OxA-failed radiocarbon samples from layer 2 (spit 2b). For taxonomic and skeletal part details, see Table S3.
(3.03 MB TIF)

Figure S9 Pego do Diabo: microlith recovered in sediments from profile collapse accumulated at the bottom of the 1960s trench. The scale is in mm. Although described as a “small microgravette point made of red flint,” and used to support a Gravettian age for layer 2 of Pego do Diabo [55], this microlith is neither made of red flint nor a microgravette. Such elongated segments (or fusiform bipoints) are entirely unknown in the Gravettian of Portugal (none have been recorded among a total of 3291 retouched tools from 15 assemblages [54]). They are not inconsistent with a Mesolithic age, but are of a type that is common in the Neolithic; the freshness and lack of patina are also consistent with a later Holocene age. The item may belong in the funerary context explored by the excavators of the 1960s trench, otherwise represented by the fragmentary human remains recovered in 1988–89 from layers 1 and A of the adjacent squares (see Tables S4–S5).
(2.80 MB TIF)

Figure S10 “Dufour bladelets”: Pego do Diabo vs. Portuguese Gravettian. Scatter plot of width versus thickness for the dataset in Table S6 (Dufour bladelets defined inclusively, as in the type-list [57], i.e., subsuming bladelets with only marginal, direct retouch).
(0.25 MB TIF)
