# Supplementary material for: Pego do Diabo (Loures, Portugal): Dating the Emergence of Anatomical Modernity in Westernmost Eurasia
Source: PLoS One. 2010 Jan 27;5(1):e8880. doi: 10.1371/journal.pone.0008880 (PMC2811729; doi:10.1371/journal.pone.0008880)
Supplement: Table S1 — Pego do Diabo: rabbit material in layer 3 radiocarbon sample ICEN-491 (a). (0.10 MB PDF) [file pone.0008880.s001.pdf]

Table S1 – Pego do Diabo: rabbit material in layer 3 radiocarbon sample ICEN-491 (a).

**Body part**	**N**
Skull (and fragments)	1
Loose teeth	32
Mandible (and fragments)	26
Maxilla (and fragments)	7
Vertebra	5
Sacrum	4
Rib (and fragments)	2
Scapula (proximal)	2
Humerus (distal)	12
Ulna (proximal)	8
Radius (distal)	1
Pelvis (and fragments)	52
Femur (whole)	3
Femur (proximal)	19
Femur (distal)	9
Tibia (proximal)	8
Tibia (distal)	32
Astragalus	3
Calcaneum	39
Metapodial	100
Phalanx	43
Tarsal/Carpal bone	4
Diaphysial fragments	4
Total	416

(a) Data from lab submission form; all the rabbit bones in this sample came from squares M13 and M14.
