# Supplementary material for: Pego do Diabo (Loures, Portugal): Dating the Emergence of Anatomical Modernity in Westernmost Eurasia
Source: PLoS One. 2010 Jan 27;5(1):e8880. doi: 10.1371/journal.pone.0008880 (PMC2811729; doi:10.1371/journal.pone.0008880)
Supplement: Table S2 — Pego do Diabo vs. Gruta do Caldeirão: species percentages (a). (0.12 MB PDF) [file pone.0008880.s002.pdf]

Table S2 – Pego do Diabo vs. Gruta do Caldeirão: species percentages (a).

Taxon	PGD-2 Aurignacian	PGD-1/2D Aurignacian	PGD-1/2D/2/REM Aurignacian	CAL Mousterian	CAL EUP	CAL Solutrean	CAL Magdalenian
Aurochs	-	5	–	1	-	+	2
Ibex	5	15	6	10	20	9	1
Ibex/Chamois	-	-	9	2	7	5	2
Chamois	2	3?	5	4	3	5	1
Red deer	60	28	43	41	35	56	37
Roe deer	-	3?	–	1	1	1	1
Wild boar	-	5	3	2	-	+	6
Equid	10	8	16	20	15	9	3
Hare	2	-	2	-	2	-	10
Beaver	-	-	–	+	-	-	+
Hyaena	2	+	1	5	3	-	-
Bear	2	3	1	1	8	+	-
Badger	-	15	1	-	-	3	11
Lion	-	-	–	-	2	+?	-
Leopard	2	-	–	1	-	2	2
Lynx	7	13	6	9	3	13	17
Wildcat	-	-	–	-	-	1	3
Wolf	2	3	3	2	-	+	-
Fox	5	-	3	1	1	+	5
N (excluding rabbits)	42	39	459	122	116	267	175.5
N (rabbits) (c)	(32)	(70)	707	(171)	(220)	(2209)	(2275)

(a) CAL = Gruta do Caldeirão; PGD-2 = Pego do Diabo, layer 2; PGD-1/2D = Pego do Diabo, layer 1 plus disturbed areas of layer 2. PGD-1/2D/2/REM = Pego do Diabo, layer 2 plus all other disturbed contexts whose Pleistocene faunal content was inferred to derive from layer 2 [60-61]. The numbers from [60-61] are NISP for the total assemblage. The other counts are "PoSAC" [65]. The data for Caldeirão are after [65] for the larger mammals and after [66] for the rabbits.
