# Supplementary material for: Pego do Diabo (Loures, Portugal): Dating the Emergence of Anatomical Modernity in Westernmost Eurasia
Source: PLoS One. 2010 Jan 27;5(1):e8880. doi: 10.1371/journal.pone.0008880 (PMC2811729; doi:10.1371/journal.pone.0008880)
Supplement: Table S3 — Pego do Diabo: OxA-failed AMS samples. (0.14 MB PDF) [file pone.0008880.s003.pdf]

Table S3 – Pego do Diabo: OxA-failed AMS samples.

**Square**	**Number**	**Level**	**Description**	**Age class**	**Observations**
L11	1	2 (spit 2a)	Shaft fragment	Adult	*Equus*/*Bos* size
M11	4	2 (spit 2a)	*Equus* metacarpal	Juvenile	–
M11	10	2 (spit 2a)	Second phalanx, *Cervus elaphus*	Adult	Carnivore-damaged
M11	11	2 (spit 2a)	Second phalanx, *Cervus elaphus*	Adult	–
M11	12	2 (spit 2a)	Second phalanx, *Cervus elaphus*	Adult	–
M11	16	2 (spit 2b)	First phalanx, *Cervus elaphus*	Juvenile	–
M11	17	2 (spit 2b)	Astragalus, *Cervus elaphus*	Juvenile	–
M11	21	2 (spit 2b)	Radius, *Cervus elaphus*	Juvenile	–
M11	25	2 (spit 2b)	Second phalanx, *Cervus elaphus*	Juvenile	–
M11	30	2 (spit 2b)	Tibia, *Cervus elaphus*	Juvenile	–
L11	sc33(20)	3	Shaft fragment	Adult	*Equus*/*Bos* size
L11	sc33(27)	3	Shaft fragment	Adult	*Equus*/*Bos* size
L11	sc33(29)	3	Shaft fragment	Adult	Mineralized; polished surface, with abrasive striations; manganese staining
