# Supplementary material for: Pego do Diabo (Loures, Portugal): Dating the Emergence of Anatomical Modernity in Westernmost Eurasia
Source: PLoS One. 2010 Jan 27;5(1):e8880. doi: 10.1371/journal.pone.0008880 (PMC2811729; doi:10.1371/journal.pone.0008880)
Supplement: Table S4 — Pego do Diabo human skeletal remains. (0.13 MB PDF) [file pone.0008880.s004.pdf]

Table S4 – Pego do Diabo human skeletal remains.

Square	Field #	Layer	Description	Age class (a)	Individual
IN SQUARES ADJACENT TO THE 1960s TRENCH (b)					
M14	–	A2	Lower left canine	Adult, dental wear 3	Adult 1
M13	sc10	2top	Third phalanx	Adult, dental wear 6	Adult 2
M13	sc28	2top	Lower lateral incisor right, worn	Adult, dental wear 6	Adult 2
J12	1	2top	Lower left first molar (1/3 root development)	Infant 1B (2-7 years)	Infant 3 (~4 years)
J12	2	2top	Juvenile right scapula	Infant 1B (2-7 years)	Infant 4 (~5-6 years)
J12	sc24	3 (c)	Intermediate hand phalanx	Adult	–
L-M11	35	1	Lower right third molar	Adult, dental wear 6	Adult 2
IN SEDIMENTS FROM PROFILE COLLAPSE AT THE BOTTOM OF THE 1960s TRENCH					
REM1	–	–	Proximal hand phalanx	Infant 1A (0-2 years)	Infant 1 (~1 year)
REM1	–	–	Upper premolar fragment	Adult, dental wear 3	Adult 1
REM1	–	–	Metcarpal diaphysis	Infant 1A (0-2 years)	Infant 1 (~1 year)
REM1	–	–	Distal epiphysis of radius	Infant 1B (2-7 years)	Infant 4 (~5-6 years)
REM1	17	–	Lower left first molar, wear 1	Infant 1B (2-7 years)	Infant 4 (~5-6 years)
REM1	32	–	Deciduous upper right first incisor, wear with distinct dentine hairline	Infant 1B (2-7 years)	Infant 2 (~3 years)
REM2	–	–	Upper first molar right, level 6 of wear	Adult, dental wear 6	Adult 2
IN SQUARES AT THE ENTRANCE OF THE CAVE (d)					
M9	sc1	2D	Proximal hand phalanx	Infant 1B (2-7 years)	Infant 3 (~4 years)
M7	sc1	2D	Skull vault fragment	Infant 1A (0-2 years)	Infant 1 (~1 year)
M6	sc21	2D	Lower second molar left, level 6 of wear	Adult, dental wear 6	Adult 2
M6	sc26?	2D	Lower incisor, only labial half	Adult, dental wear 3	Adult 1
N6	sc6	2D	Lower left first molar, no wear	Infant 1B (2-7 years)	Infant 2 (~3 years)
N6	sc8	2D	Distal hand phalanx, proximal ½ preserved	Adult	–
L-M5	sc8	2D	Lower right second incisor, wear 3	Adult, dental wear 3	Adult 1
L-M5	sc9	2D	Proximal hand phalanx	Infant 1B (2-7 years)	Infant 4 (~5-6 years)
L-M5	sc10	2D	Proximal hand phalanx	Infant 1B (2-7 years)	Infant 4 (~5-6 years)
L-M5	sc11	2D	Middle hand phalanx	Adult	–
L-M5	sc19	2D	Thoracic vertebra	Adult	–
L-M5	sc31	2D	Deciduous left upper canine, significant wear	Infant 1B (2-7 years)	Infant 4 (~5-6 years)
L-M5	sc23	2D	Parietal fragment with lambda?	Infant 1A (0-2 years)	Infant 1 (~1 year)
L-M5	sc33	2D	Incisor, anterior half, with calculus	Adult	–
L-M5	sc44	2D	Lower left premolar (P4)	Adult, dental wear 3	Adult 1

(a) After [106].

(b) Together with the material from profile collapse recovered at the bottom of the 1960s trench, this scatter of small-sized skeletal elements probably reflects the existence in squares L-M12 of a Holocene funerary context explored (and nearly exhausted) by the unknown excavators of that trench.

(c) J12 corresponded to a small remnant preserved against the north wall of the cave that was almost entirely disturbed by a burrow that extended down to the interface between layers 2 and 3, explaining the presence of this human phalanx, intrusive from the Holocene burial context documented by the material recovered in layers A and 1.

(d) This cluster of small and fragmented material, mostly recovered in squares 5-6 of the grid, right at the entrance, where the sediment fill was only ca.20 cm thick on average, very probably corresponds to sieving or backdirt residue from the trench excavated at the back of the cave in the 1960s.
