# Supplementary material for: Pego do Diabo (Loures, Portugal): Dating the Emergence of Anatomical Modernity in Westernmost Eurasia
Source: PLoS One. 2010 Jan 27;5(1):e8880. doi: 10.1371/journal.pone.0008880 (PMC2811729; doi:10.1371/journal.pone.0008880)
Supplement: Table S5 — Pego do Diabo: AMS radiocarbon results obtained for human skeletal remains (a). (0.12 MB PDF) [file pone.0008880.s005.pdf]

Table S5 – Pego do Diabo: AMS radiocarbon results obtained for human skeletal remains (a).

**Sample**	**Lab #**	**Age [^{14}C years BP]**	**cal BC age**	**Used [g]**	**Yield [mg]**	**Yield [%]**	**C [%]**	**C:N [ratio]**	**δ^{13}C [‰]**	**δ^{15}N [‰]**
J12-2	VERA-4981	4115±45	2880-2570	0.55	10.3	1.9	39 (c)	3.1 (c)	-18.6 ± 0.8 (b)<br>-20.0 (c)	7.4 (c)
J12sc24	VERA-4982	4490±35	3350-3030	0.72	13,3	1.8	43 (c)	3.2 (c)	-15.9 ± 2.9 (b)<br>-19.4 (c)	8.2 (c)
	VERA-4982UF1	4525±40	–	0.48	2.0	0.4	–	–	-19.1 ± 1.0 (b)	–
	VERA-4982UF2	4485±45	–	–	4.0	0.8	–	–	-20.6 ± 0.8 (b)	–

a) Pretreatments: standard gelatin production and ultrafiltration (UF1 result – gelatin fraction >30 kDa; UF2 result – gelatin fraction <30 kDa). All uncertainties in radiocarbon age and δ^{13}C results are 1σ. Calibration used OxCal 3.10 with the INTCAL04 calibration curve, and cal BC results are the 95.4 % probability age ranges.

b) Determined with the AMS system in the graphitized sample.

c) Determined via EA-IRMS measurement in a gelatin portion split from the ^{14}C dated gelatin [EA-IRMS measurement precision of a repeatedly measured standard material: 0.1‰ (1 σ) for δ^{15}N and δ^{13}C].
