# Supplementary material for: Pego do Diabo (Loures, Portugal): Dating the Emergence of Anatomical Modernity in Westernmost Eurasia
Source: PLoS One. 2010 Jan 27;5(1):e8880. doi: 10.1371/journal.pone.0008880 (PMC2811729; doi:10.1371/journal.pone.0008880)
Supplement: Table S6 — Pego do Diabo vs. Portuguese Gravettian: relative frequencies of bladelet tool categories (a). (0.12 MB PDF) [file pone.0008880.s006.pdf]

Table S6 – Pego do Diabo vs. Portuguese Gravettian: relative frequencies of bladelet tool categories (a).

Assemblages	Dufour bladelets		Bladelets with marginal, direct retouch		Backed bladelets and points		Geometrics	
	N	%	N	%	N	%	N	%
AURIGNACIAN								
Pego do Diabo, layers 2+2D	6	86	1	14	–	–	–	–
EARLIER GRAVETTIAN (b)								
Vale Comprido-Barraca	3	12	1	4	21	84	–	–
Fonte Santa	–	–	–	–	4	40	6 (c)	60
LATER GRAVETTIAN (b)								
Terra do Manuel 1940-42	13	5	7	3	234	91	2 (d)	1
Cabeço de Porto Marinho II, lower inf	–	–	–	–	17	100	–	–
Terra do Manuel 1988-89, layer 2s	1	7	–	–	13	93	–	–
Cabeço de Porto Marinho III, middle	1	12	–	–	7	88	–	–

(a) Only assemblages with more than five pieces in the bladelet tool category [41, 54].

(b) In the Gravettian assemblages, the "Dufour bladelet" category is totally comprised of elements whose inverse retouch is marginal, none featuring the kind of semi-abrupt, invasive retouch applied to the ventral side of the alternate-retouched Aurignacian Dufours from Pego do Diabo.

(c) Includes three small fragments of curved, backed pieces classified as segments that probably correspond in fact to broken backed-curved microlithic points, a type represented in the assemblage by a complete specimen of the same module (L=22.3 mm; W=8.5 mm; Th=3.5 mm); curved-backed microlithic point types of identical module are also represented by three specimens in the Early Gravettian from Vale Comprido-Barraca, and by four specimens in the Late Gravettian from Terra do Manuel (three) and Terra do José Pereira (one).

(d) Includes one small segment (L=24.1 mm; W=8.1 mm; Th=2.7 mm) that may well belong in the Neolithic context otherwise represented in the assemblage by a small polished adze, a broken bone pendant, a potsherd and a transversal arrowhead (all probably surface finds, as suggested by similar material from layer 1—the plough zone—of the 1988-89 excavations).
