# Supplementary material for: Pego do Diabo (Loures, Portugal): Dating the Emergence of Anatomical Modernity in Westernmost Eurasia
Source: PLoS One. 2010 Jan 27;5(1):e8880. doi: 10.1371/journal.pone.0008880 (PMC2811729; doi:10.1371/journal.pone.0008880)
Supplement: Table S7 — Taphonomically reliable 14C and TL results for the Middle-to-Upper Paleolithic transition in Iberia (a). (0.16 MB PDF) [file pone.0008880.s007.pdf]

Table S7 – Taphonomically reliable ^{14}C and TL results for the Middle-to-Upper Paleolithic transition in Iberia (a).

Site	Layer	Composition	Method	Lab #	Age [^{14}C years BP]	cal BP age
LATEST MIDDLE PALEOLITHIC						
Sima de las Palomas	2e	Burnt bone	AMS ^{14}C	OxA-10666	34,450±600	41,650-37,730
	2l	Burnt rabbit bone	AMS ^{14}C	OxA-15423	35,030±270	41,880-38,400
Cueva Antón (b)	I-k	*Pinus nigra* charcoal	AMS ^{14}C	OxA-21244	32,890±200	38,670-35,750
Jarama VI	2.2	Charcoal	AMS ^{14}C	Beta-56639	32,600±1800	42,080-33,040
	2.1	Charcoal	AMS ^{14}C	Beta-56638	29,500±2700	40,200-28,720
Gorham's Cave	Trench 7 m inside from main area	*Pinus* sp. charcoal	AMS ^{14}C	OxA-10295	34,600±900	42,010-37,170
	IV from back of the cave	*Pinus* sp. charcoal, cone scale	AMS ^{14}C	OxA-10230	32,330±390	38,590-34,950
	Context 24	Charcoal	AMS ^{14}C	OxA-7857	32,280±420	38,580-34,860
Gruta da Oliveira	8	Burnt bone	AMS ^{14}C	GrA-10200	31,900±200	36,320-35,200
		Burnt bone	AMS ^{14}C	OxA-8671	32,740±420	38,760-35,440
EARLIEST UPPER PALEOLITHIC						
Cueva Bajondillo (c)	11	Sediments & charcoal	AMS ^{14}C	Ua-17150	33,690±1195	42,210-34,770
		Sediments & charcoal	AMS ^{14}C	Ua-18050	32,770±1065	40,520-34,400
Gato Preto (d)	C	Burnt flint	TL	British Museum	–	45,900-30,300
Cueva de Mallaetes (e)	XII	Charcoal	^{14}C	KN-I/926	29,690±560	34,890-32,930
Gorham's Cave (f)	Context 9	*Pinus* sp. charcoal	AMS ^{14}C	OxA-7076	30,250±700	35,700-33,220
		Pinus sp. charcoal	AMS ^{14}C	OxA-7074	30,200±700	35,650-33,170
		Pinus sp. charcoal	AMS ^{14}C	OxA-7075	29,800±700	35,210-32,770
		Pinus sp. charcoal	AMS ^{14}C	OxA-7077	29,250±650	34,730-32,370
	Context 11	Burnt bone	AMS ^{14}C	OxA-7388	29,100±340	34,320-32,760
	Context 13a	*Pinus* sp. charcoal	AMS ^{14}C	OxA-7110	29,250±750	34,850-32,210
	Context 15	Charcoal	AMS ^{14}C	OxA-7792	28,680±240	33,860-32,460
	Layer D (Waechter's excavation)	Charcoal	^{14}C	GrN-1455	28,700±200	33,830-32,550
		Charcoal	^{14}C	GrN-1363	27,860±300	33,020-31,780

(a) After [19, 50, 107]. Calibration used CalPal with the CalPal_2007_HULU calibration curve [33-34], and cal BP results are the 95.4 % probability age ranges.

(b) ABOx-SC method. Two samples from this level treated with the normal ABA method yielded a younger result (~31.1 ka ^{14}C BP in both cases), indicating that residual contaminants could not have been removed by standard pre-treatment techniques, a problem that may explain <32 ka ^{14}C BP charcoal results for other sites of the Iberian Middle Paleolithic.

(c) Because the site is a rock shelter excavated in Middle-Upper Pleistocene travertines, the inclusion of sediment in these samples may have contaminated them with unknown amounts of old carbon; therefore, they should be treated as maximum ages only.

(d) Probability interval calculated from the average (38100±3900 BP) of the results obtained for samples GPR4 and GPR11.

(e) The lithics are not diagnostic, but a bone point from this layer is typical of the later Aurignacian (II or III-IV).

(f) The lack of index fossils precludes assignation of the diagnostically Upper Paleolithic stone tool assemblages recovered from these Gorham's Cave contexts to either the Aurignacian II or the Aurignacian III-IV, but the dating places them firmly within the range of the later Aurignacian and excludes other blade-based technocomplexes of the Iberian Upper Paleolithic (Gravettian and Solutrean).
