# Supplementary figures and images for: Pego do Diabo (Loures, Portugal): Dating the Emergence of Anatomical Modernity in Westernmost Eurasia
Source: PLoS One. 2010 Jan 27;5(1):e8880. doi: 10.1371/journal.pone.0008880 (PMC2811729; doi:10.1371/journal.pone.0008880)

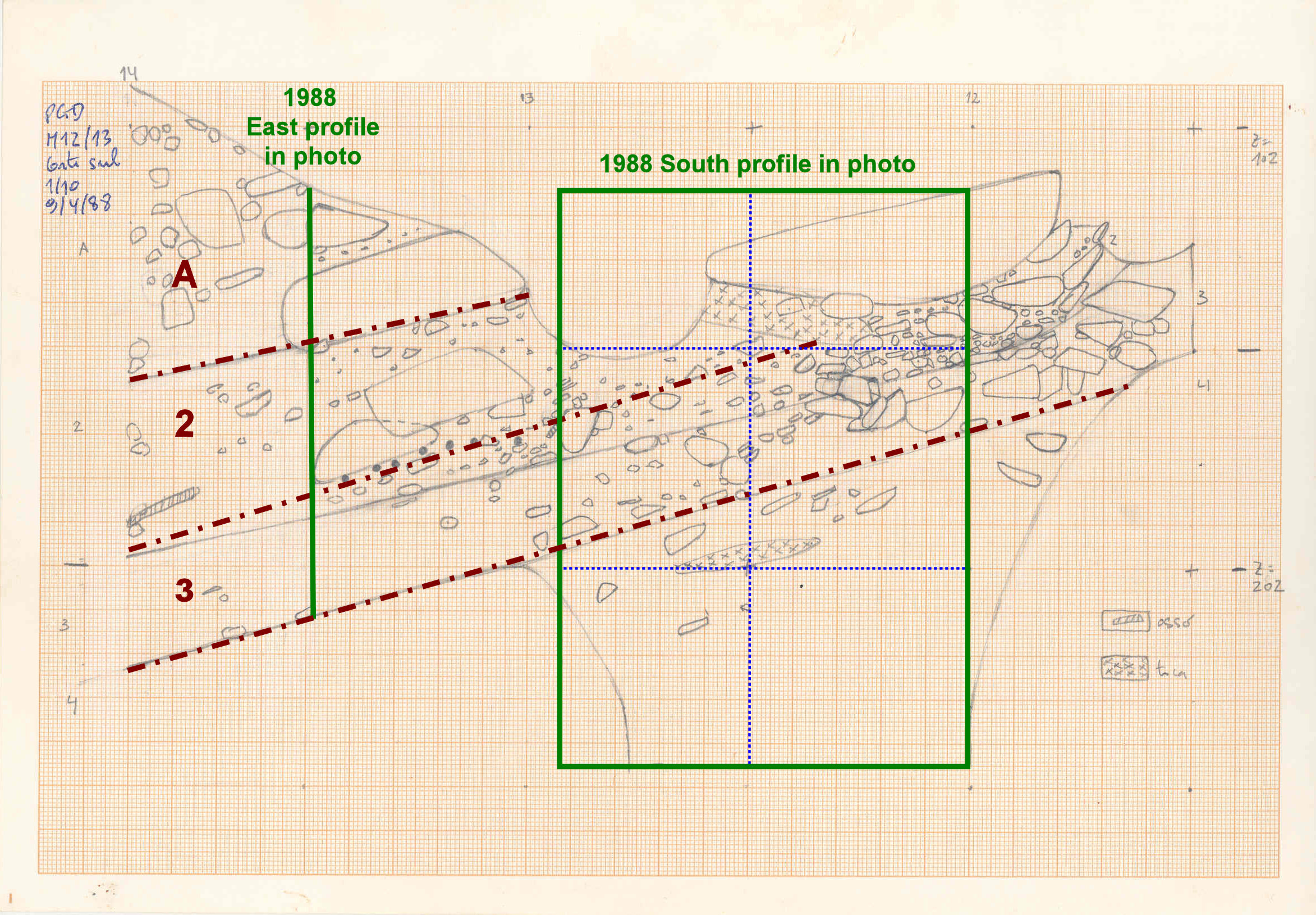

Supplement: Figure S1 — Pego do Diabo: M>N14W-12 profile. Facsimile reproduction of the field drawing, with indication of the position of the profiles whose photographs are given in Figure 5. Note the post-excavation correction of the boundary between layers 2 and 3. This rectification has no implication for the assignment of finds from the 1988–89 field work, as it affects an area where the profile results from the excavation of the 1960s trench. (9.37 MB TIF) [file pone.0008880.s008.tif]

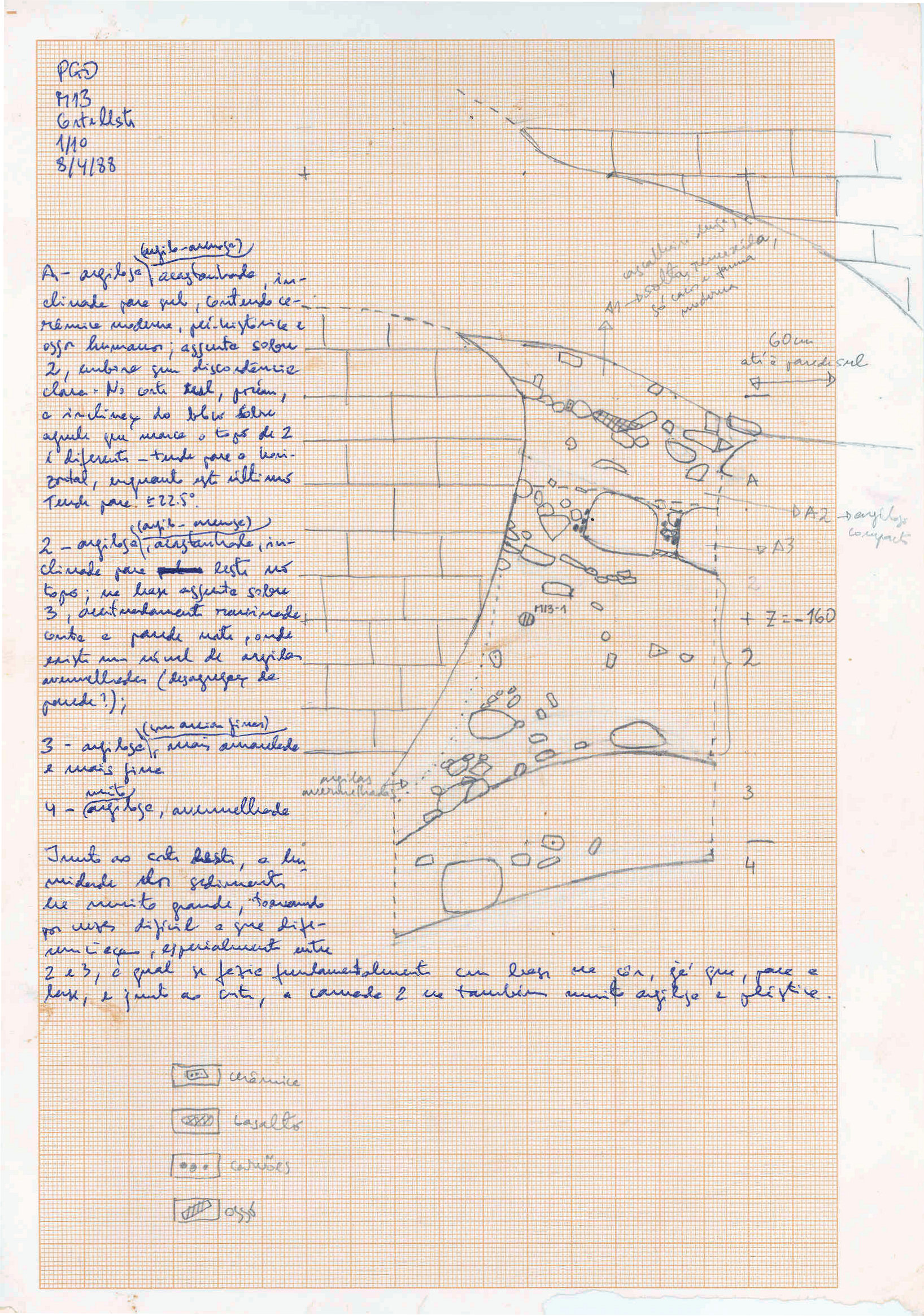

Supplement: Figure S2 — Pego do Diabo: M13>14 profile. Facsimile reproduction of the field drawing. (9.71 MB TIF) [file pone.0008880.s009.tif]

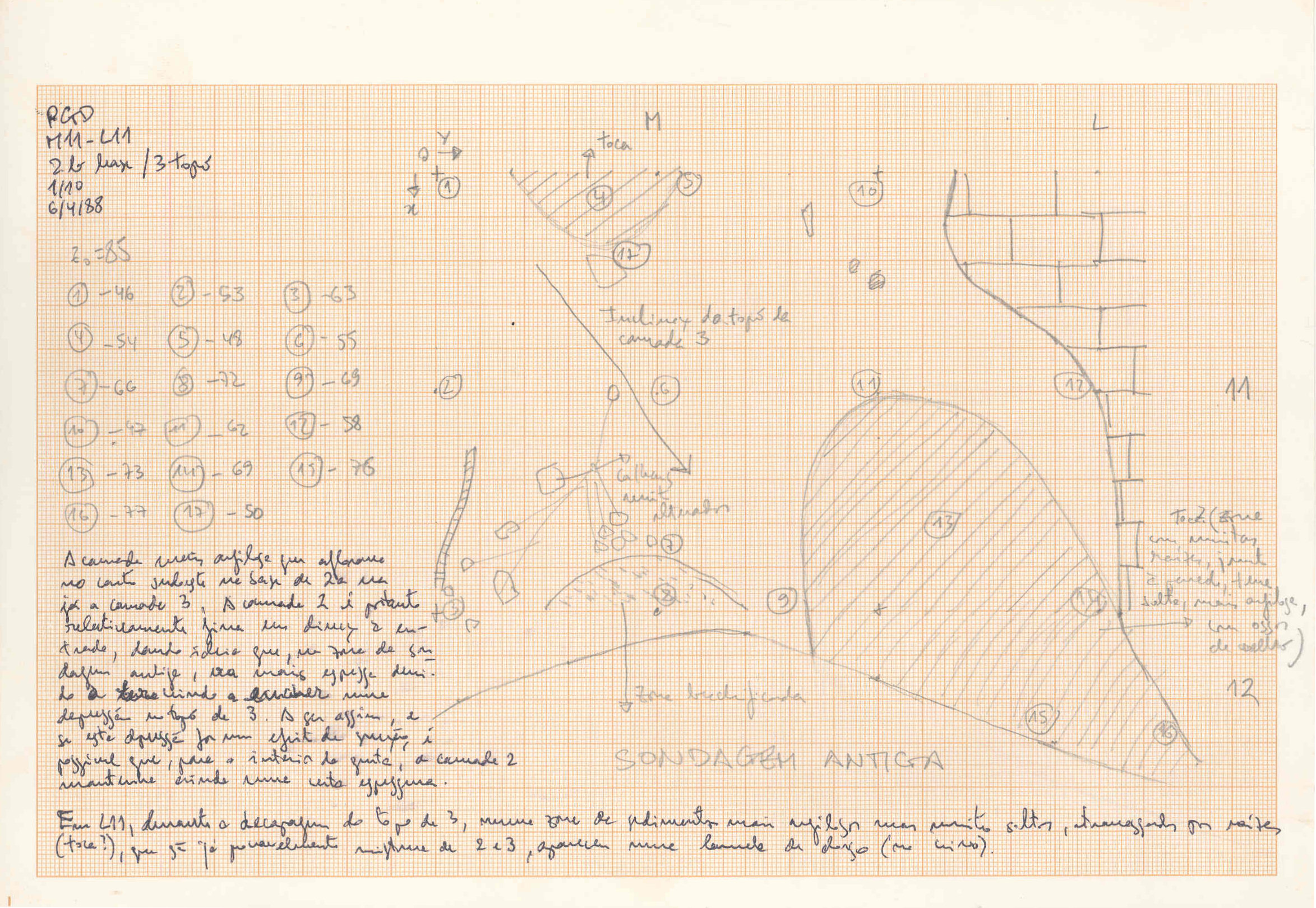

Supplement: Figure S3 — Pego do Diabo: the surface of layer 3 in squares L-M11. Facsimile reproduction of the field drawing. (9.05 MB TIF) [file pone.0008880.s010.tif]

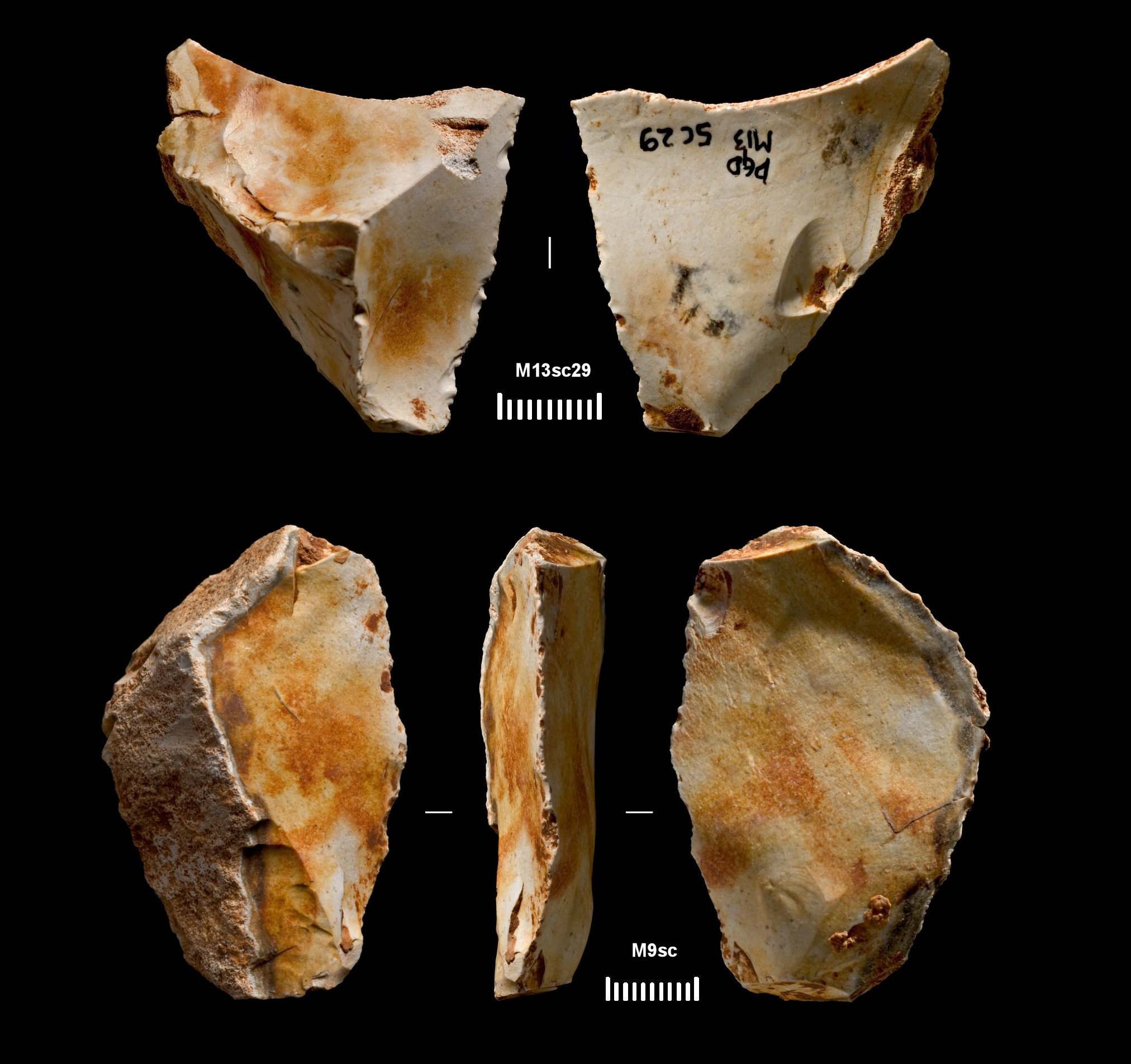

Supplement: Figure S4 — Pego do Diabo: lithics from layers 3–4. Top: flake M13sc29, from layer 3. Bottom: flake from layer 4, square M9. Note the patina and edge damage, which suggest that the accumulation of these and the few other flints of similar appearance recovered in layers 3–4 relates to natural inwash processes, not to human activity at the site. In 1965 or 1966, a group from the Palethnology Department of the Portuguese Speleological Society collected flint flakes in ploughed fields immediately above the limestone ridge where the cave opens (Carl Harpsøe, personal communication, March 07, 2009), and such may well be the provenience of the lithics found in these layers. (3.05 MB TIF) [file pone.0008880.s011.tif]

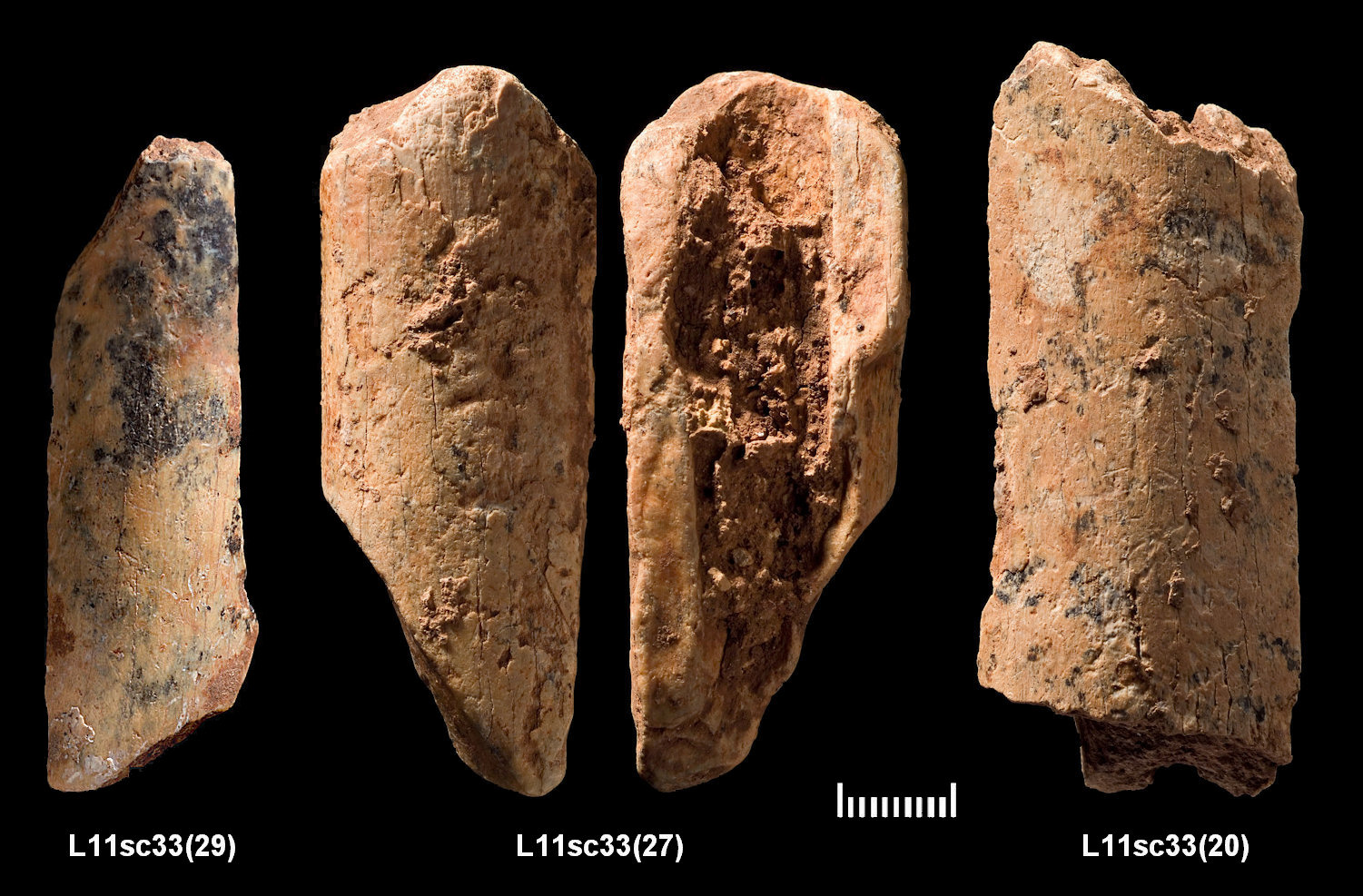

Supplement: Figure S5 — Pego do Diabo: failed radiocarbon samples from layer 3. All come from square L11 (see Table S3 for further details). Note the manganese staining. (2.27 MB TIF) [file pone.0008880.s012.tif]

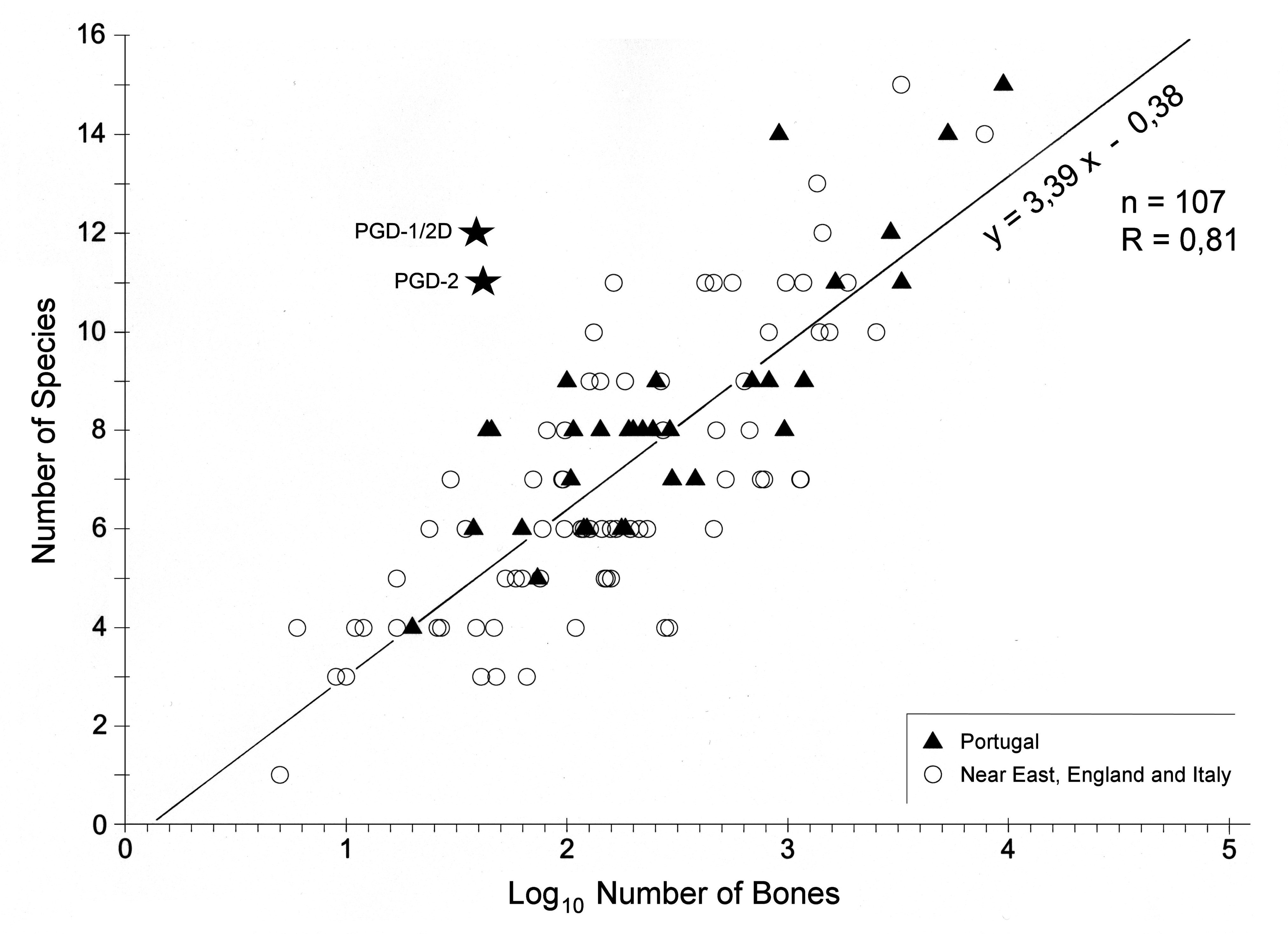

Supplement: Figure S6 — Pego do Diabo: taxonomic diversity in context. A plot of the number of mammal species (of size equal to or greater than a rabbit) against the decimal logarithm of the number of bones of mammals (of size equal to or greater than a rabbit) identified to species level from 107 archeological sites/levels in Europe and the Near East studied with the PoSAC method [65], [98]. Sheep and goat are treated as a single taxon as are the various species of equids. The numbers of bones range from 5 to 9673 and the numbers of species range from 1 to 15. The abundance of taxa observed in layer 2 of Pego do Diabo is all the more striking because of the small size of the mammal bone assemblage. (1.44 MB TIF) [file pone.0008880.s013.tif]

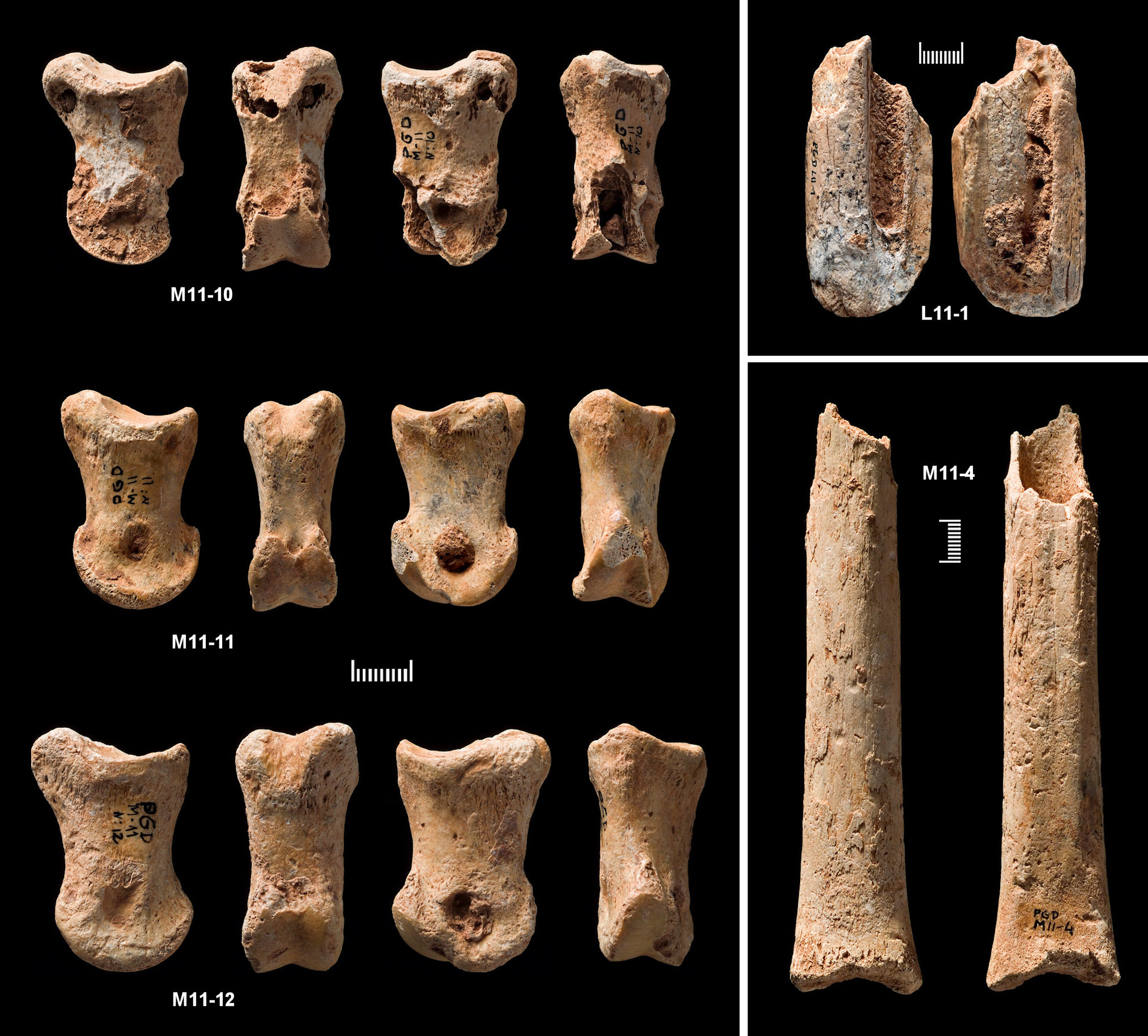

Supplement: Figure S7 — Pego do Diabo: OxA-failed radiocarbon samples from layer 2 (spit 2a). For taxonomic and skeletal part details, see Table S3. (4.31 MB TIF) [file pone.0008880.s014.tif]

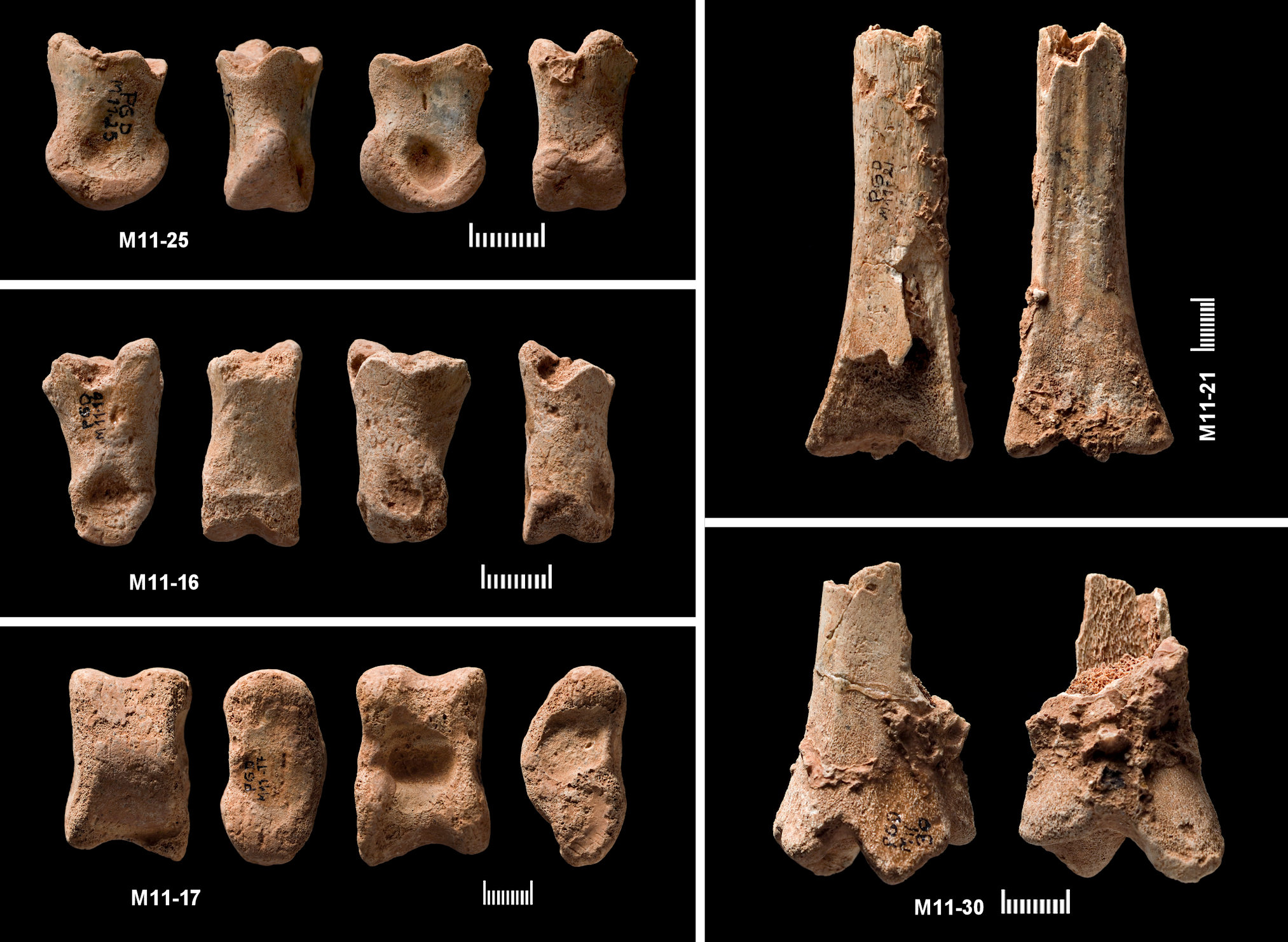

Supplement: Figure S8 — Pego do Diabo: OxA-failed radiocarbon samples from layer 2 (spit 2b). For taxonomic and skeletal part details, see Table S3. (3.03 MB TIF) [file pone.0008880.s015.tif]

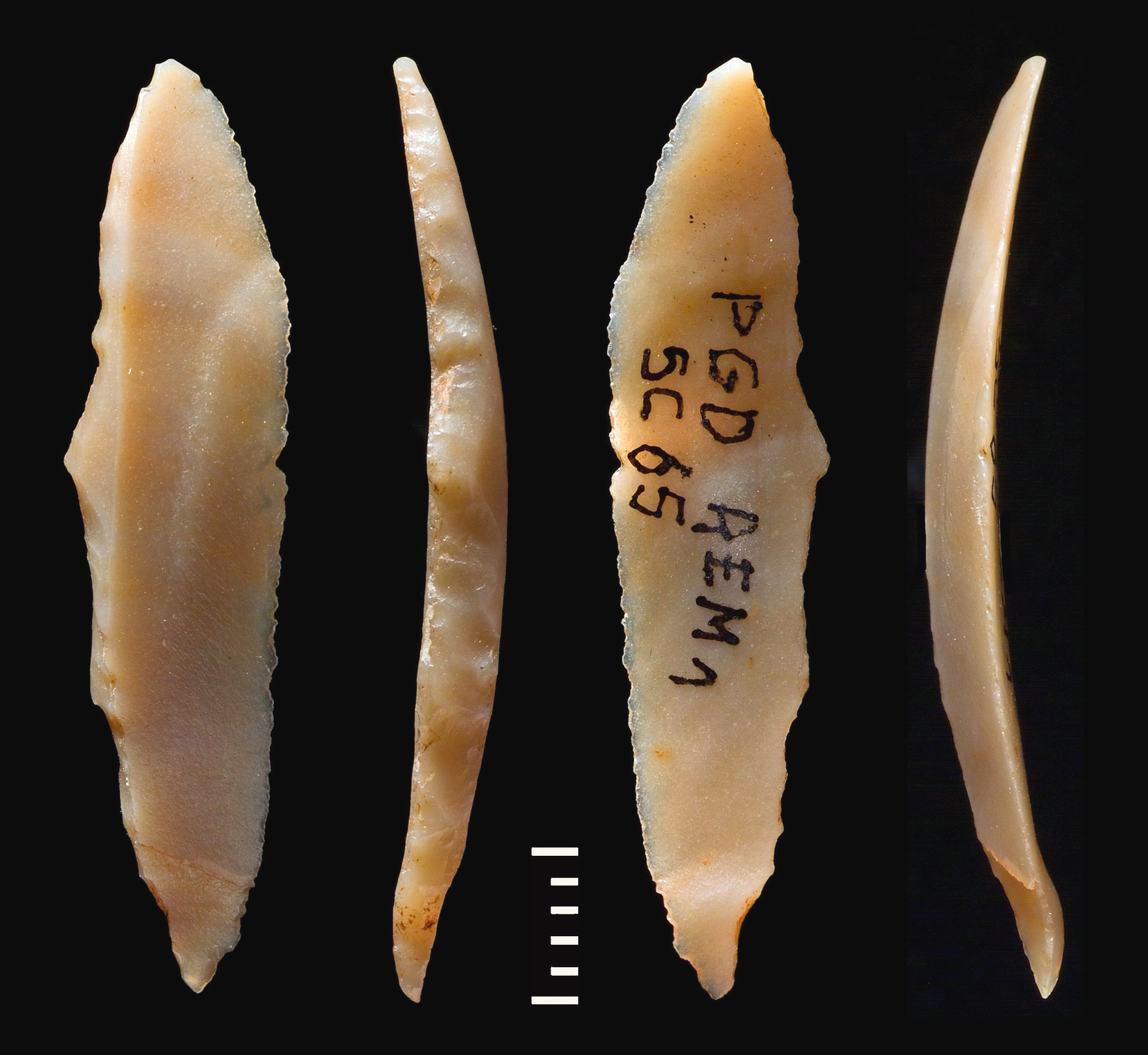

Supplement: Figure S9 — Pego do Diabo: microlith recovered in sediments from profile collapse accumulated at the bottom of the 1960s trench. The scale is in mm. Although described as a “small microgravette point made of red flint,” and used to support a Gravettian age for layer 2 of Pego do Diabo [55], this microlith is neither made of red flint nor a microgravette. Such elongated segments (or fusiform bipoints) are entirely unknown in the Gravettian of Portugal (none have been recorded among a total of 3291 retouched tools from 15 assemblages [54]). They are not inconsistent with a Mesolithic age, but are of a type that is common in the Neolithic; the freshness and lack of patina are also consistent with a later Holocene age. The item may belong in the funerary context explored by the excavators of the 1960s trench, otherwise represented by the fragmentary human remains recovered in 1988–89 from layers 1 and A of the adjacent squares (see Tables S4–S5). (2.80 MB TIF) [file pone.0008880.s016.tif]

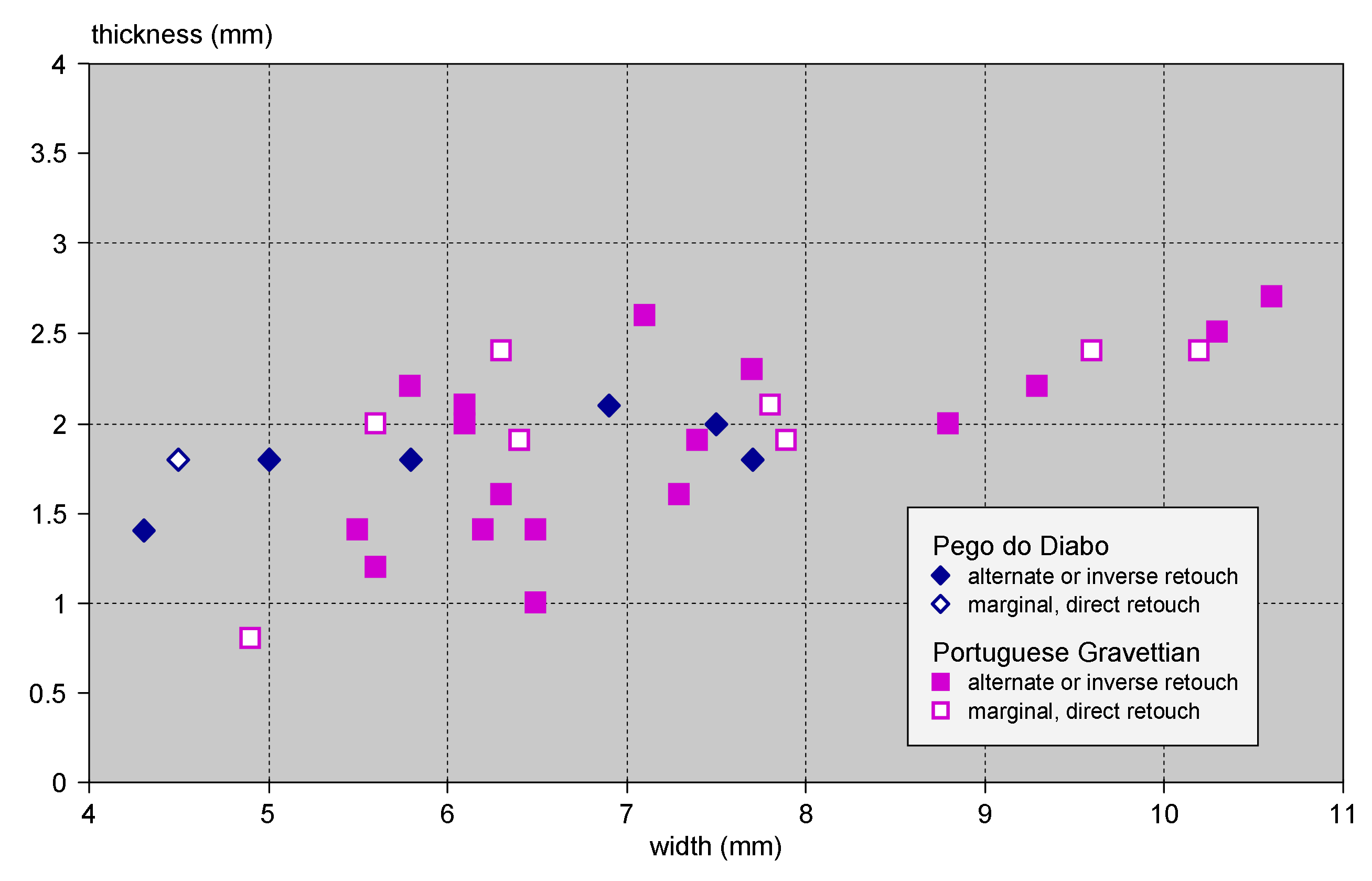

Supplement: Figure S10 — “Dufour bladelets”: Pego do Diabo vs. Portuguese Gravettian. Scatter plot of width versus thickness for the dataset in Table S6 (Dufour bladelets defined inclusively, as in the type-list [57], i.e., subsuming bladelets with only marginal, direct retouch). (0.25 MB TIF) [file pone.0008880.s017.tif]
